# Biomimetic Flexible Sensors and Their Applications in Human Health Detection

**DOI:** 10.3390/biomimetics8030293

**Published:** 2023-07-06

**Authors:** Huiwen Yu, Hao Li, Xidi Sun, Lijia Pan

**Affiliations:** Collaborative Innovation Center of Advanced Microstructures, School of Electronic Science and Engineering, Nanjing University, Nanjing 210093, China

**Keywords:** bionic, sensing, flexible, preparation, human health, electronic skin

## Abstract

Bionic flexible sensors are a new type of biosensor with high sensitivity, selectivity, stability, and reliability to achieve detection in complex natural and physiological environments. They provide efficient, energy-saving and convenient applications in medical monitoring and diagnosis, environmental monitoring, and detection and identification. Combining sensor devices with flexible substrates to imitate flexible structures in living organisms, thus enabling the detection of various physiological signals, has become a hot topic of interest. In the field of human health detection, the application of bionic flexible sensors is flourishing and will evolve into patient-centric diagnosis and treatment in the future of healthcare. In this review, we provide an up-to-date overview of bionic flexible devices for human health detection applications and a comprehensive summary of the research progress and potential of flexible sensors. First, we evaluate the working mechanisms of different classes of bionic flexible sensors, describing the selection and fabrication of bionic flexible materials and their excellent electrochemical properties; then, we introduce some interesting applications for monitoring physical, electrophysiological, chemical, and biological signals according to more segmented health fields (e.g., medical diagnosis, rehabilitation assistance, and sports monitoring). We conclude with a summary of the advantages of current results and the challenges and possible future developments.

## 1. Introduction

In the 1960s, a new comprehensive discipline of bionics emerged internationally, which is based on biology, electronics, biophysics, and automation technology. It uses electronics and mechanical technology to study biological structures or to simulate the processes of energy conversion and information flow. As people pay more attention to health, more and more people are focusing on the field of health monitoring. Among them, micro/nanoelectronic devices, especially flexible sensors, with thin and portable, high sensitivity, good biosafety, excellent electrical performance, and high integration [1], can enhance the role of smart medicine in disease diagnosis and treatment, motion monitoring, and health prevention, which has become the focus of medical service work.

With the rapid development of microelectronics science and technology, the requirements of the manufacturing process of microelectronic devices are also increasing [2], and the flexibility of sensors is being improved to reduce the discomfort caused by these devices and to reduce the psychological and physical burden of users. Flexible electronics are electronic technologies that fabricate electronic devices on flexible or ductile plastic or thin metal substrates. They can achieve stretchability and bendability without damaging their own electronic properties, and with their excellent ability to adapt to deformation, they greatly expand the scope of application of traditional inorganic electronic devices.

Among the bionic structural devices targeting human physiological signals, skin-like surface devices, represented by electronic skin that imitates human skin properties and other functions, have been widely studied. As an extremely important internal and external interaction organ of the human body, skin devices can find their applications in the implementation of various functions such as pressure, temperature, distance, pain, and friction [3,4,5,6,7,8,9]. As research has evolved, researchers have also realized that they should not limit themselves to imitating the structure and multifunctional sensing capabilities of human skin but should also develop excellent properties beyond skin [10,11,12]. Physiological information monitoring devices inspired by natural organisms and their specific organ functions have also been developed in the field of medical health for many years, and in recent years, there have also been studies by researchers to implement these bionic efforts on flexible sensors. In this paper, we will present the current role played by flexible sensor devices and the current state of development in various perspectives and fields of human health, using bionic as a clue to summarize and explore the prospects and new challenges of bionic flexible sensors in the health field.

As people pay more attention to health, more and more people start to pay attention to the field of health monitoring. The bionic flexible sensor, as a new type of device, has the characteristics of thinness and portability, high sensitivity, good biosafety, excellent electrical performance, and high integration, which enhance the role of intelligent medicine in disease monitoring and early warning, and has just become the focus of current medical service work. This paper will firstly introduce the definition and sensing mode of bionic flexible sensors, secondly summarize the advanced materials for preparing bionic sensors, and finally, introduce and prospect the bionic flexible sensors and their applications in the field of human health monitoring.

## 2. Basic Principles of Bionic Flexible Sensors

### 2.1. Common Signal Types

Bionic flexible sensing devices were developed based on a new detection method that detects micro-signals from the body surface or bio-active materials such as body fluids and enzymes during physiological activities. Bionic is the design and improvement of the sensor and the process of giving the sensor certain unique biological properties by studying and using the working principles and structure of living organisms. Flexible sensors are sensors made of flexible materials, including substrates, with flexible and diverse structural forms, pursuing good flexibility and ductility, and can be arbitrarily arranged according to the requirements of measurement conditions, making them suitable for complex application scenarios or objects.

Depending on the type of physiological signals collected, bionic sensors can be classified as physical signal bionic sensors and chemical signal bionic sensors. Among the various signals present in nature, the presence of force, temperature, and humidity is felt from time to time as essential and important for our normal life, providing signals for changes in the environment and physiological state [13,14]. This will be the basic content of physical signal sensors (force, temperature or humidity sensors, etc.) discussed in this review. In addition, there is a special class of electrical form signals, biopotentials, that are present in the activity of organs, tissues, and neurons. By monitoring electrocardiograms (ECG) [15,16,17,18,19], electroencephalograms (EEG) [18,20], electromyograms (EMG) [18,19,21], and electroencephalograms (EOG) [18,22,23], these electrophysiologic (EP) signals can be used for early diagnosis of disease and management of chronic conditions such as arrhythmias, myocardial infarction, and epilepsy.

**Figure 1:**
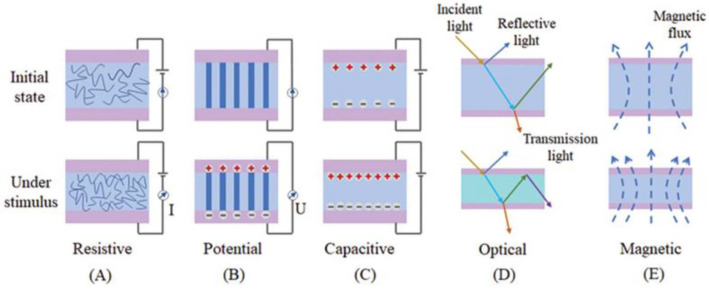
Schematic diagram of common physical sensing mechanisms [47]. Copyright 2021, John Wiley and sons. (**A**) resistive sensing mechanisms, such as piezo-resistive, thermo-resistive, etc.; (**B**) potentiometric sensing mechanisms, such as piezoelectric, thermoelectric, frictional-electric, etc.; (**C**) capacitive sensing mechanisms, such as piezoelectric-capacitive, etc.; (**D**) optical sensing; (**E**) magnetic sensing mechanisms.

The operating mechanisms of physical signal sensors include electrical (resistance-capacitance and potential), optical, and magnetic mechanisms [24,25]. When subdivided, one part is divided into conduction mechanisms for strain and pressure sensing: piezoresistive [26,27,28], piezoelectric [29,30], frictional electrical [31], and capacitive [32,33,34] effects. Another part is temperature sensing mechanisms: thermal resistance (thermosensitivity), thermoelectricity, thermal capacitance, and thermal expansion effects [29,35,36,37], while humidity sensing mechanisms are generally determined through the resistance and capacitance changes caused by the material in response to an external stimulus [38,39]. For bionic flexible sensors to convert external stimuli into measurable signals, they can be broadly divided into electrical responses (resistance, potential, and capacitance signals) [40] and non-electrical signals (e.g., optical or magnetic signals) [36,41,42]. Today, the former dominates and is widely reported in the literature, while the latter often serves as an important complement to enrich the types of response signals available to sensors (Figure 1).

These instruments are characterized by low detection limits, wide measurement ranges, high resolution, and ultra-fast response times. However, they also have potential disadvantages, such as thermoelectric/thermal and piezoelectric/triboelectric sensors, which can only respond to dynamic temperature or pressure stimuli and cannot monitor static and quasi-static stimuli [43,44]. In the case of capacitive sensors, changes in temperature can cause changes in capacitance due to changes in the geometric volume and internal structure of the sensitive material. In addition, changes in temperature also affect the transport capacity of charge carriers such as ions and electrons, changing the relative capacitance and capacitance [45]. Similarly, due to the interaction between water molecules and moisture-sensitive materials, when the relative humidity changes, the water molecules interact with the moisture-sensitive active material, causing structural changes and mechanical deformation, which affects the mobility of charge carriers and thus changes the dielectric constant. Thus, capacitive strain sensors have an advantage over resistive sensors in terms of accuracy and stability of sensory signals and dependence on temperature/humidity [46]. However, this advantage turns into a disadvantage that seriously affects their performance when not used as such.

#### 2.1.1. Pressure and Strain Sensors

Pressure sensors are the “old-timers” of the sensor world. They can be found in many health applications, such as health and exercise monitoring, repair of human sensory organs, and implantable devices. Pressure and strain sensors are typically based on the conversion of electrical signals, such as changes in the three electrical fundamentals of impedance, current, and voltage caused by external stimuli. Pressure and strain are detected using conduction principles such as capacitive, piezoresistive, piezoelectric, and frictional electricity. Of these, capacitive and piezoresistive sensors are the most commonly used because of their simple design and their ability to detect both static and dynamic signals [48]. Their design principles must be differentiated according to the type of strain stimulus. Piezoresistive sensors typically rely on changes in the path of current flow through the conductive material in the polymer matrix or changes in the contact resistance between the piezoresistive material and the electrodes to detect pressure or strain [49] because they can convert mechanical strain into electrical signals such as open-circuit voltage (VOC) and short-circuit current (ISC) [50,51]. Most capacitive sensors are characterized by a dielectric layer sandwiched between two electrodes to form a “sandwich” structure, and when pressure or strain is applied, changes in the distance between the electrodes and the area of the device occur due to geometric deformation or changes in the effective dielectric constant of the dielectric layer, which can result in changes in capacitance between the electrodes. The main parameters that affect strain or pressure sensors are sensitivity, dynamic range, response, and recovery time. Using bionics, we can develop dielectric layers with high performance and biocompatibility for pressure sensors, such as plant leaves [52,53,54], human tissue [55,56,57], abrasive paper [58,59,60], etc.

#### 2.1.2. Temperature Sensor

Although body temperature can vary slightly depending on environmental temperature conditions or physical activity, human body temperature is broadly regulated to remain within a constant temperature range of approximately 36.5–37.5 °C, which is essential for the enzymatic activity involved in metabolism, circulation, and the immune system [61]. Irregular and abnormal shifts in body temperature are, in some cases, considered indicators of disease (acute fever and hypothermia). Therefore, regular monitoring of changes in body temperature is an important application of health monitoring. Due to the extremely narrow range of normal temperature variations in the human body (close to 1 °C) [62], temperature sensors for healthcare monitoring require high resolution, accuracy, responsive sensitivity, and a targeted detection range.

In order to develop flexible, wearable, and highly sensitive temperature sensors, some efforts are already underway on thermally responsive materials and temperature sensing methods. The response of thermally responsive materials to the action of temperature signals is reflected in changes in their electrical and optical properties. Several types of sensing methods have been proposed to quantify temperature changes, such as thermistors [63,64], thermocouples [65], thermoelectric effects [66], and colorimetric methods [67,68] (visualization of temperature changes). Thermistors are classified as having either a negative or positive temperature coefficient, depending on whether they exhibit a decrease or increase in resistance with temperature, respectively, with negative temperature coefficients being the most commonly used because their effect on resistance is more in line with the logic of a positive correlation of response with temperature. Piezoelectric materials contain pyroelectric materials, which in turn contain ferroelectric materials [47]. Thus, ferroelectric materials can produce three types of effects: ferroelectric, thermoelectric, and piezoelectric [69]. Devices composed of thermoelectric materials can achieve both temperature and pressure sensing [70].

**Figure 2:**
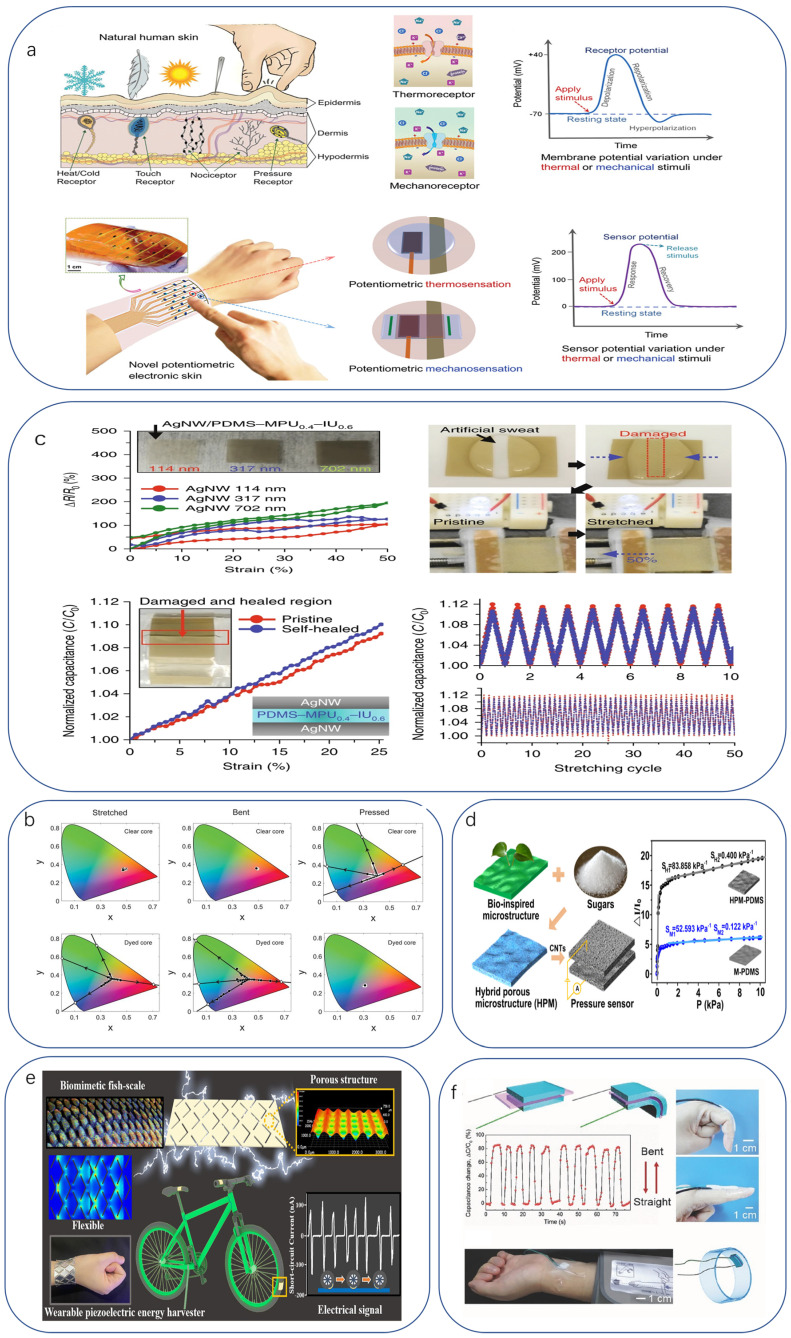
(**a**) Bionic design of potentiometric e-skin for thermal and mechanical sensing [35]. Copyright 2021, John Wiley and sons. (**b**) Chromatic response of transparent core (top) and chromatic response of colored core (bottom) when SLIMS is stretched, bent, or strained in the colored region [81]. Copyright 2020, The American Association for the Advancement of science. (**c**) Interconnects and sensors with autonomous self-healing capabilities [89]. Copyright 2018, Springer Nature. (**d**) Highly sensitive flexible piezoresistive pressure developed using bionic textured porous material sensors [193]. Copyright 2019, American Chemical Society. (**e**) Combination of solid-state shear milling and fused wire fabrication 3D printing strategies to fabricate high-performance bionic wearable fish-scale PVDF-based piezoelectric energy harvesters [198]. Copyright 2022, American Chemical Society. (**f**) Bio-inspired mineral-based self-healing hydrogel sensors for finger motion monitoring and blood pressure sensing [199]. Copyright 2017, John Wiley and sons.

The human epidermis is a biological pyroelectric and piezoelectric material that can sense temperature and pressure by pyroelectric and piezoelectric means [71]. Therefore, further research on electronic skin lies in exploring the chemical and physical basis and sensing mechanisms of human skin. For example, Arias et al. fabricated a biomimetic bifunctional e-skin [35] via an ion-conducting PVA/NaCl/Gly electrolyte system that can both compute heat in the form of potentials and sense mechanical actions, but there is still room for refinement by mainly monitoring static and slowly changing thermal and force stimuli (Figure 2a). Although the discussion is about temperature detection on the human body and does not over emphasize this drawback, bionic flexible temperature sensors often have materials that do not tolerate temperature well, allowing the devices to be prepared for optimal performance in a limited low-temperature range. This goes against the conventional perception that thermocouple devices are linear and durable for hazardous and harsh environments.

#### 2.1.3. Chemical Sensors

Electrolytes, metabolites, and hormones are abundant in biofluids. Chemical sensors detect and analyze these biomarkers and extract important physiological information from them at the molecular level [72,73], again enabling early diagnosis and prevention of diseases. Therefore, in the last few years, many research efforts have been performed on skin-attachable chemosensors for medical purposes [49].

Once a chemical sensor (mainly a sensing electrode) comes into contact with a target chemical, the device typically exhibits a change in potential (for charged analytes), current (for redox-active analytes), or resistance. For example, potential sensors typically use ion-selective electrodes that are designed to respond to a specific target analyte; compound resistance is the sensing element between two electrodes that changes when exposed to the target analyte. Finally, considering transistor-based chemosensor, which consist of a semiconductor layer, a dielectric layer, and three electrodes (source, drain, and gate), they can be thought of as adding a dielectric layer and a gate electrode to a chemoresistor, with transistors having a well-known special ability to achieve signal amplification and high sensitivity [49].

Chemical sensors can detect very low concentrations of chemicals, typically in the ppm (parts per million) or ppb (parts per billion) level. Chemical sensors are designed to be sensitive only to a specific chemical or class of chemicals, making them excellent representatives of this property of selectivity, but different substances with similar functional groups and similar chemical properties also potentially threaten this property. Chemical sensors can provide results in seconds and can therefore be used for real-time monitoring, but some chemical sensors can lose their ability to function due to factors such as ambient temperature and humidity. When the sensor fails and degrades, it will face the challenge of biochemical contamination.

There are still many factors to consider when using chemical sensors in practical medical applications. For example, human body fluids contain low concentrations of chemicals and have a large number of substances with similar chemical compositions; therefore, chemical sensors for practical applications should have high selectivity, low detection limits, high sensitivity, and high reproducibility.

#### 2.1.4. Light/Magnetic Sensors

In addition to the above transduction mechanisms, there are non-electrical signals, such as optical signals, which are triggered by mechanisms such as mechanical luminescence (photoexcitation) [74,75], thermochromism [76], electroluminescence [77], physical properties of light (absorption, diffraction, scattering, and so on), and changes in the reflectance of the active material in question [78,79]. Factors such as strain, temperature, and humidity can cause changes in the reflectivity of optical materials, so GE uses monolithic muscle-inspired self-healing and antifreeze hydrogel strain and temperature sensors with positive and negative resistive responses, respectively [80]. It clearly displays four different colors when the joint is bent, stretched, or compressed, and the optical path in the fiber changes under different deformations/forces [81] (Figure 2b). A typical feature of optical sensors is their fast response time, typically in the nanosecond range, which allows them to be used in high-speed applications (e.g., fiber optics) with a certain level of accuracy. However, to ensure the same response speed and accuracy, the target light source/light signal must be strong enough to operate, and it is obvious that extraneous light sources in the environment can cause interference and are difficult to isolate.

Magnetic sensors operate on the principle of electromagnetic induction, which converts the measured object (such as vibration, displacement, speed, etc.) into a sensor of induced electric potential through magneto-electric interactions. When the magnetic field changes, the magnetic sensor generates a corresponding electrical signal, allowing the magnetic field to be detected. Magnetic sensors are widely used in modern industry and electronic products to induce magnetic field strength to measure physical parameters such as current, position, and direction and are most commonly associated with health monitoring, namely MRI. However, current devices used for human magnetic monitoring rely on a strong power supply to generate electromagnetic fields of sufficient strength and range, which makes changing the number of components, size, and flexibility of key sensors still a major challenge.

In general, three basic components are required to monitor or detect the various signals mentioned above, namely the sensor/active element, the substrate, and the conductive electrode [47].

### 2.2. Common Materials for Bionic Flexible Sensors

#### 2.2.1. Substrates

The substrate is the most fundamental design element of the device, which demonstrates the relevant bionic structure, such as the various types of artificial tentacles, cilia, and scale surfaces, precisely through the surface morphology and distribution structure of the substrate; the sensing function depends on the choice of material and the usage scheme. While plastic-based materials have excellent mechanical and electrical stability and robustness, their performance in terms of stretchability does not match the scenario of human applications. In contrast, elastomers, which are inherently stretchable, are more attractive because they maintain excellent mechanical properties both in bending and folding and in kneading and wrinkling [82]; PDMS, Ecoflex, and TPU are examples of this [83,84,85]. It is also based on the application of elastomeric substrates that Zhenan Bao proposed the concept of e-skin, which physically mimics the elasticity, stretchability, and softness of skin [49,86,87].

Due to their flexibility, ductility, light weight, and low cost, polymer films are often used as substrates for flexible sensors, mainly made from polymers such as polyester (PET), polyimide (PI), polyetherimide (PEI), p-xylene, or polyethylene naphthalene dicarboxylate (PEN). Of the rubber-based materials, polydimethylsiloxane (PDMS) is particularly noteworthy. It is the most commercially available material and has been shown to have good research properties, so it is widely used in the manufacture of electronic skins and other flexible electronics. Its advantages include chemical inertness, temperature stability, and variable mechanical properties, as well as the ability to achieve both viscous and nonviscous region preparation under UV exposure conditions [88]. For example, Wang et al. reported a stretchable and self-healing e-skin based on a PDMS-MPU_0.4_-IU_0.6_ substrate with stretch rates up to 1600%. The fabricated e-skin can withstand up to 100% strain without damage and can even repair itself after damage [89] (Figure 2c). Other different types of flexible materials such as Ecoflex [90,91], natural rubber [92], etc., thermoplastic elastomers such as SBS [93], POE [94,95], etc., dynamically cross-linked polyimide [96], hydrogels [97,98] (e.g., PVA), and cellulose [99,100], are good candidates for flexible substrates.

Despite the above advantages of elastomer-based wearable sensors, it is important to note that they do not perform well in cycling tests in terms of electrical stability and reliability due to aging, hysteresis, and the fact that some rubber chains break under rapid stretching [101,102]. In addition, most sensors made of either plastic or elastomer-based materials do not perform well in terms of permeability, leading to user discomfort and poor adhesion. Some work has therefore extended the choice of materials to fabrics made of natural or synthetic polymers, such as cotton, silk, nylon, etc., which, as described, have good elasticity and breathability and are also a good choice for device preparation. This idea of applying functional materials to the fibers or sewing functional fibers into the fabric substrate [103] is the proposed e-textile concept.

#### 2.2.2. Electrode Circuit Materials

Current electrodes and interconnect circuits for flexible electronic devices are typically made of metals, conductive polymers, carbon, and liquid metals [82]. Researchers have used ultrathin conductive path designs in the form of serpentine, fractal, and kirigami structures [104,105] to enhance the otherwise flexible and limited properties of solid metals. The advantage of this design is that it is compatible with conventional micro- and nanofabrication processes while providing high conductivity. However, the fabrication process is still complex, and the stretchability is naturally and unavoidably limited (by geometry and bulk material properties). Conductive polymers are currently a promising class of material candidates. Although conventional conductive polymers also exhibit stiffness and extensibility of approximately 5% [104,106], it is possible to modify this property through additive or modified solutions. Stretchability can be greatly improved by the addition of nonionic and ionic small molecules [107] and by binding to elastomers or hydrogels [108]. Flexible conductive wires can be fabricated by dispersing carbon and metal microparticles in the form of carbon nanotubes, graphene, metal nanoplatelets, and microfilaments into elastomers [109]. As a further application of this technique, these complexes of conductive particles and polymers are available as conductive inks for 3D or screen printing, greatly reducing the cost of patterning them into interconnected networks. The disadvantage of this approach is that the conductivity of the composite is not as high as that of a pure metal wire because the polymer is not conductive. In addition, due to the presence of rigid fillers, the upper limit of strain that the mechanical properties of the composite can withstand does not yet cover the vast majority of applications [82]. To this end, a better material solution is being sought, such as liquid metals [110,111]. For example, eutectic gallium–indium alloy (EGaIn), which has good intrinsic strain and conductivity equivalent to metallic conductivity—very low piezoresistivity—is considered a promising material. It combines with polymers or elastomers to form high-performance flexible conductors that reach new heights of conductivity and mechanical properties [112]; liquid metal/polymer composites are also less costly to produce for printing inks. However, it is not without significant drawbacks, as it exists as a liquid component and is subject to the risk of leakage, which limits the reliability of stretchable devices. In this regard, embedding liquid metals in elastomers can be a good alternative, although the high surface tension properties of liquid metals and the spontaneous oxidation reactions on the material surface pose a challenge to their patterning [49].

#### 2.2.3. Active Materials

Active materials include carbon nanomaterials (CB, CNT, graphene, GO, and rGO) [113,114,115], metal nanowires [116] or nanoparticles [117,118,119], liquid metals [120,121,122,123], conductive polymers [124,125,126], ionic liquids [123,127,128], etc. The choice of materials for the sensing active element is based on the operating principle. In flexible bioelectric sensors, the sensing active materials range from conductors and semiconductors to dielectrics for the transduction mechanism employed [82]. In general, resistive [129,130], dielectric [131,132], piezoelectric [133], and friction [134,135] materials are used for the sensing active components, such as poly(3-hexylthiophene) (P3HT): [6,6]-phenyl-C61-butyric acid methyl ester (PCBM) mixtures or peroxides [136].

Most functional nanomaterials are sensitive to moisture, such as carbon nanomaterials, MXene, AgNWs, conductive polymers, and some water-sensitive polymers (polyethylene glycol, polyvinyl alcohol, poly-N-isopropylacrylamide, cellulose, chitin, etc.) [47]. The basic principle is also that they undergo changes in resistance or capacitance when in contact with water [137,138,139], so they can be used as sensing active materials to indicate changes in ambient humidity or water content of articles [99,140,141]. It should be noted that the capacitance exhibited by the materials is frequency dependent, so the calculated capacitance sensitivity for relative humidity sensing will be different at different frequencies [47].

### 2.3. Preparation of Bionic Flexible Sensors

To achieve flexible sensors, materials and processes must be matched. For example, plastics, paper, and rubber are used as flexible substrates to avoid organic solvents, high temperatures, or high-powered processes that would easily destroy the materials. A key point for flexible devices that mimic human skin is that human skin stretches/contracts with body movement without damage [142]. Therefore, stretching strategies are not a negligible consideration in research: intrinsic stretchability and geometric structuring.

Geometric Engineering: The approach to brittle electronic materials to develop their stretchability can be geometric design and patterning. The so-called geometric designs, represented by wavy/snake-like structures, wrinkled structures, lattice patterns, and 3D porous structures, improve the stretchability of metallic thin films and conductive nanomaterials. These structures can minimize the damage induced by direct-acting strains in conductive materials due to the effective relaxation of external tensile stresses by such structural elongation and geometrical changes with the guiding idea of strain space reservation. Therefore, we can see many reports on the use of such curved structural patterns as wavy and pleated, as described above, applied to the development of stretchable electronics [143,144,145,146].

Intrinsic stretchability: Unlike the rigid materials actively considered for improvement in the previous section, some organic materials have their own low-cost and simple solution processing methods and are inherently stretchable. These advantages contribute to their high degree of durability and plasticity. However, the electrical properties of stretchable organic materials are poor because of the higher energy required for free electron movement due to the general characteristics of their chemical structure [49]. Current research is attempting to overcome this limitation, and it is gratifying to note that some developments have been made in recent years in the direction of improved electrical properties [147,148,149,150,151].

Single or multiple-process hybrid routes have been used to fabricate flexible sensors using conventional or novel design methods to achieve the effective structures described above. These fabrication methods include photolithography [152,153,154], stencil guiding [155,156], spraying [4], vacuum filtration [157,158], spin coating [12,32,50,159], squeegee [77,160], self-assembly [55,161], physical/chemical vapor deposition (PVD/CVD) [162,163], laser cutting [44,164], electrospinning [165], printing (e.g., screen printing, transfer printing, 3D printing, etc.) [166,167,168,169,170], and solution methods [171,172,173] (as summarized in Table 1). For example, inkjet printing technology was originally introduced for office printing applications but has since become a valuable tool for research applications and microstructure fabrication. Both piezoelectric ink deposition [174] and thermal inkjet [175] essentially use a dispenser to deposit small amounts of liquid material onto a substrate. The main advantage of this technique is the ability to reliably handle minute amounts of material (down to a few picoliter volumes of droplets). In addition, device fabrication using this technique is very economical in the use of ink formulations and can be widely applied to prototyping and low-volume manufacturing because inkjet printing technology is a maskless process where the material is deposited directly, and the desired pattern can be easily adjusted by refining the digital print file. The combination of these advantages reduces material waste; office-like equipment that does not require specialized skills and laboratories [176] has made it a common process in recent research [49,109,168,171]. This method can already be seen in medical research efforts, such as the fabrication technique combining inkjet printing and microwave irradiation described by Malekghasemi et al. [177]. Without any heating, cutting, or use of a photoresist, a hydrophobic layer formed from hexamethyldisilazane and tetraethylorthosilicate precursors on filter paper is used, which is eroded by HCL deposited by the inkjet printer. It is much simpler compared to standard microchip fabrication strategies; in particular, the use of (fibrous) paper as the substrate for the sensor platform further demonstrates the value of the process for simple layouts and inexpensive device components for sensor design concepts. The resulting device was tested for urease activity and showed the ability to detect up to 0.5 units of urease within 3 min.

Work on flexible stretchability also continues to be of interest in the preparation segment, and one way to create stretchable electronic devices is to have all electronic components be of a type with intrinsic stretchability (either in the preparation material or in the processing method) [189]. For example, matrix circuits based on Ti/Au nanofilms fabricated by electron beam deposition are being used for strain and humidity sensing of highly sensitive electronic skins [190].

The use of microstructures is a common processing approach to improve the performance of flexible pressure sensors [24,26,191,192]. This is more common in bionic work because, with the exception of mimicking intrinsic biological and physiological activity, most of the entry points and explanatory theories for functional bionics can be made from the specificity that the structure of their physiological tissue/organ has, which can be designed by advanced fabrication techniques with specific resemblance patterns of flexible sensors to achieve the desired sensing performance. Especially for resistive and piezoelectric devices, the introduction of micro-patterned structures in the fabrication process can increase the sensitivity and is therefore widely used [193] (Figure 2d). When micro/nanopattern designs are to be constructed in the dielectric layer, they are usually grown by stencil-induced growth or by standard lithography methods. Conventional micropattern designs use geometries such as pyramids, columns, and spheres, which can either be arranged in neat arrays or have different sizes and distributions throughout the dielectric layer as needed [194,195]. The sensing principle of the dielectric layer was described earlier as essentially a variation in electrode spacing and device area, and patterning is useful here as well. Larger local stress concentrations lead to different expressions of the contact area variation curve, thereby modulating the sensitivity range accordingly [196]. As a specific application of this approach, bionic patterning works in much the same way. In addition, the fabrication of flexible transistor-based devices can also be used to monitor physical and physiological signals [98,197]. The mechanism by which this works, i.e., the output of VOC and ISC in response to mechanical stimuli, as described above, is also an important complementary piece.

Finally, we discuss the limitations of many current processes that were developed in the past with the goal of preparing flat devices. This is not conducive to focusing on adhesion when the object is our three-dimensional body due to its irregular surface profile and wide range of energy deformation. We have summarized three patterning strategies to adapt to this challenge [49]. The first is to continue to use traditional printing or lithography techniques to create flat, flexible devices that are then transferred to a 3D surface, which obviously involves many steps, is highly restrictive to the transfer process, and has predictably poor conformability. Another technique is to use 3D printing. For example, PEI has developed a 3D-printed bionic wearable energy harvester inspired by environmentally adaptive fish-scale materials that can harvest energy through various deformations and motions. The energy harvester is a 3D-printed bionic wearable energy harvester that can harvest energy through various deformations and motions (Figure 2e) [173]. The third technique is a return to traditional large-area coating techniques, such as brushing or spraying, where sensing materials are applied to a 3D surface. However, it can only process one type of sensing material at a time. For example, Wu coated a bio-inspired mineral-based self-healing hydrogel sensor that can detect pressure and strain [174] (Figure 2f).

## 3. Bionic Flexible Sensors for Human Health Monitoring Applications

### 3.1. Bionic Flexible Sensors in Medical Diagnosis and Treatment

The most basic method for the diagnosis of disease and discomfort is the quantitative analysis of physical indicators to determine its magnitude in relation to prescribed values, commonly represented by, for example, invasive sampling by blood sampling, non-invasive sampling by urinalysis, and electro-physical signal acquisition based on large instruments. These methods highlight the disadvantages of high harm, weak privacy, and poor mobility. In contrast, flexible and especially bionic functional/structure-based diagnostic devices can further improve the reliability, sensitivity, and biosafety of the devices while ameliorating these drawbacks. This can be reflected by: non-invasive/low-invasive acquisition and timely result feedback, relying on simple biological structures to achieve high sensitivity to specific physiological substances or physical signals, giving the device more monitoring functions than conventional types, improving the suitability of the device to the internal physiological environment of the human body, reducing the risk of rejection and traumatic infection, and developing devices that conform to physiological principles to facilitate clinical studies on humans.

#### 3.1.1. Diagnostic Tools

**Figure 3:**
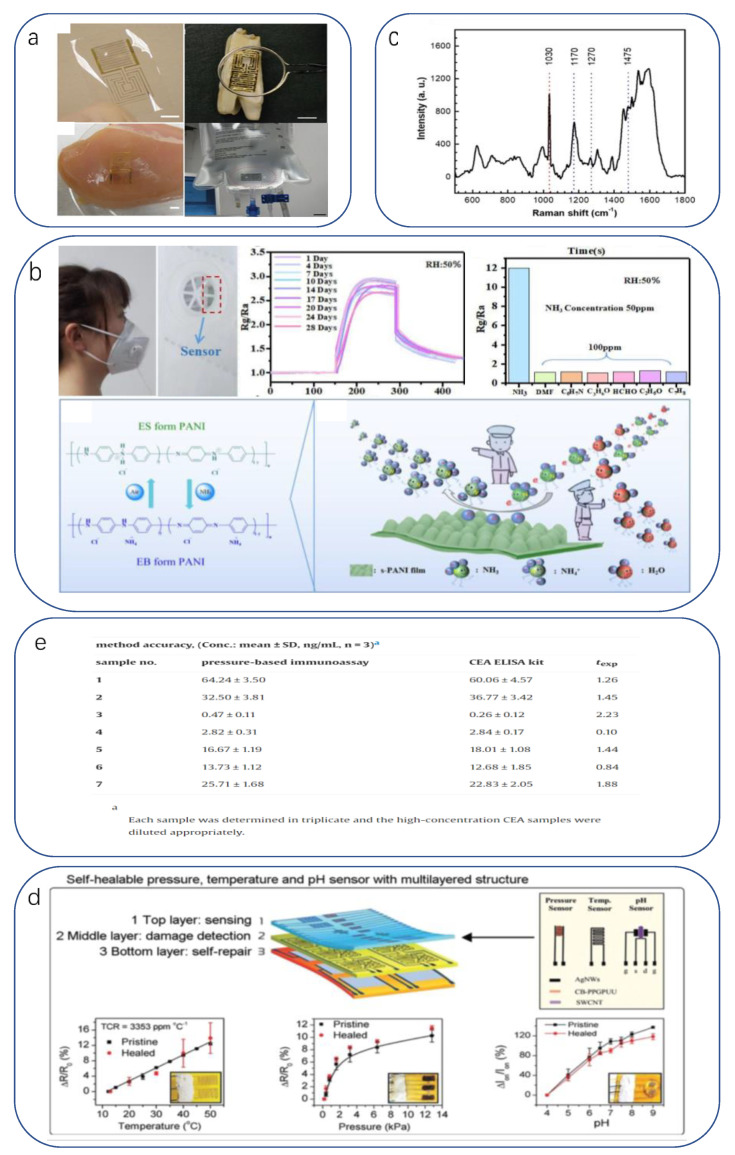
(**a**) Graphene nanosensors and their transfer to bovine molars, muscle tissue, and IV bags [200]. Copyright 2012, Springer Nature. (**b**) Characterization of the performance of flexible sensors for NH3 gas sensing [201]. Copyright 2023, Elsevier. (**c**) SERS spectra of human sweat after exercise [206]. Copyright 2019, American Chemical Society. (**d**) Self-repairable electronic skin based on multilayer multi-pixel structure for pressure, temperature and pH sensing [206]. Copyright 2020, John Wiley and sons. (**e**) Multiscale layered design based on pressure sensors for accuracy assessment of proposed immunoassays [208]. Copyright 2023, Elsevier.

The most common diagnostic observation is the sampling of human physiological substances. On the one hand, the human body is mostly composed of water, in which a large number of substances reflecting the state of the internal physiological environment and pathological features are dissolved, making it easy to extract and having a wide diagnostic range. On the other hand, it is a less time-consuming project for a larger flow of patients. McApline reported a resistive graphene electronic skin sensor printed on a bioabsorbable silk substrate. In addition to muscle tissue, the sensor can be mounted on complex surfaces after dissolving the silk substrate (Figure 3a) [200]. For example, functionalizing the graphene surface with antimicrobial peptides enables wireless detection of the presence of individual E. coli and Staphylococcus aureus. It can also detect H. pylori in saliva when applied to teeth. Li Xu et al. reported a low-contamination, simple method for fabricating flexible and transparent PANI films [201]. To achieve high sensitivity and moisture resistance, a flexible and transparent polyaniline (PANI) film with the unique feature of a bionic rose micro-dome structure was used. The micro-dome structure brings a significant deformation rate in addition to the hydrophobicity of the bionic structure, which makes the prepared sensor expected to achieve the unification of sensitivity, detection range, detection limit, and moisture resistance multiple requirements. The results show that it has a low detection limit of 10 ppb in response to NH3, and its sensing performance is much better than that of the PANI membrane without the bionic structure. It can be used in medical diagnosis for early diagnosis of some chronic kidney diseases by detecting human breath (Figure 3b).

Bodily fluid is the largest carrier of physiological substances in the human body, and the analysis of the content of these physiological substances allows us to infer the health status of the body. Among many types of bodily fluids, sweat is the most accessible and contains important and significant information such as electrolytes, metabolites, amino acids, proteins, and hormones [3,73,202,203]. This allows the monitoring of metabolic diseases and physiological conditions [160,204].

For example, Liu et al. fabricated a multifunctional flexible sensor using a reed leaf as a template [205]. This sensor imitated the microstructure of the reed leaf surface, a PDMS substrate with a reed leaf as a template on the surface was obtained by the double casting method, and then gold was plated on both sides of the PDMS substrate as electrodes. The sensor has a maximum pressure sensitivity of 0.6 kPa^−1^ (in the range of 0–1 kPa), a detection limit of 4.5 Pa, and a response time and recovery time in the hundred-millisecond range (180, 120 ms). Using this sensor, we can detect metabolites in sweat; for example, the researchers demonstrated SERS detection of human sweat samples (Figure 3c), which can detect metabolites such as lactic acid, fatty acids, and urea in typical bands. In addition to detecting sweat compounds, sensing basic solution properties such as pH is a novel idea. pH is a measure of the acidity of a solution and represents the relative amount of free hydrogen ions in the solution. However, there has been little progress in pH sensing in flexible strain and temperature sensing systems. Haick recently published work on pH, pressure, and temperature sensors that attempted a flexible sensor integration scheme with physical and chemical sensing capabilities [206] (Figure 3d). They used a relatively conventional concept of integrating three independent sensors into a flexible substrate to obtain a self-healing multi-sensor electronic skin, which provides guidance for designing or assembling multi-responsive sensors with minimal crosstalk between sensing functions.

Detection of cancer biomarkers is an important function that can be introduced into electronic skin. Zhang et al. used label-free SGGFET to detect prostate-specific antigens [207], a biomarker for prostate cancer. However, Lingling Huang proposed a flexible piezoresistive sensor based on micro/nanoscale bio-inspired layered interlocking structure films [208]. The film with two interlocking layers was prepared from TPU elastomer paired with polyaniline particles active conductive material with a sea urchin-like shell hollow structure. The sensor utilizes a gas-producing immunoassay reaction to determine the target carcinoembryonic antigen (CEA). The mechanism is simple: when both the catalytic substrate H_2_O_2_ and the target CEA are present, there is a significant current change and the current response curve. In contrast, only a weak pressure response and a flat current curve approaching zero were observed in the absence of either H_2_O_2_ or the target (Figure 3e).

In addition to the testing of biochemical markers, there is also the testing of physical signals in conventional medical diagnosis, which is also described here to determine what is happening in the patient’s body by analyzing the logic reflected in the physical signals.

**Figure 4:**
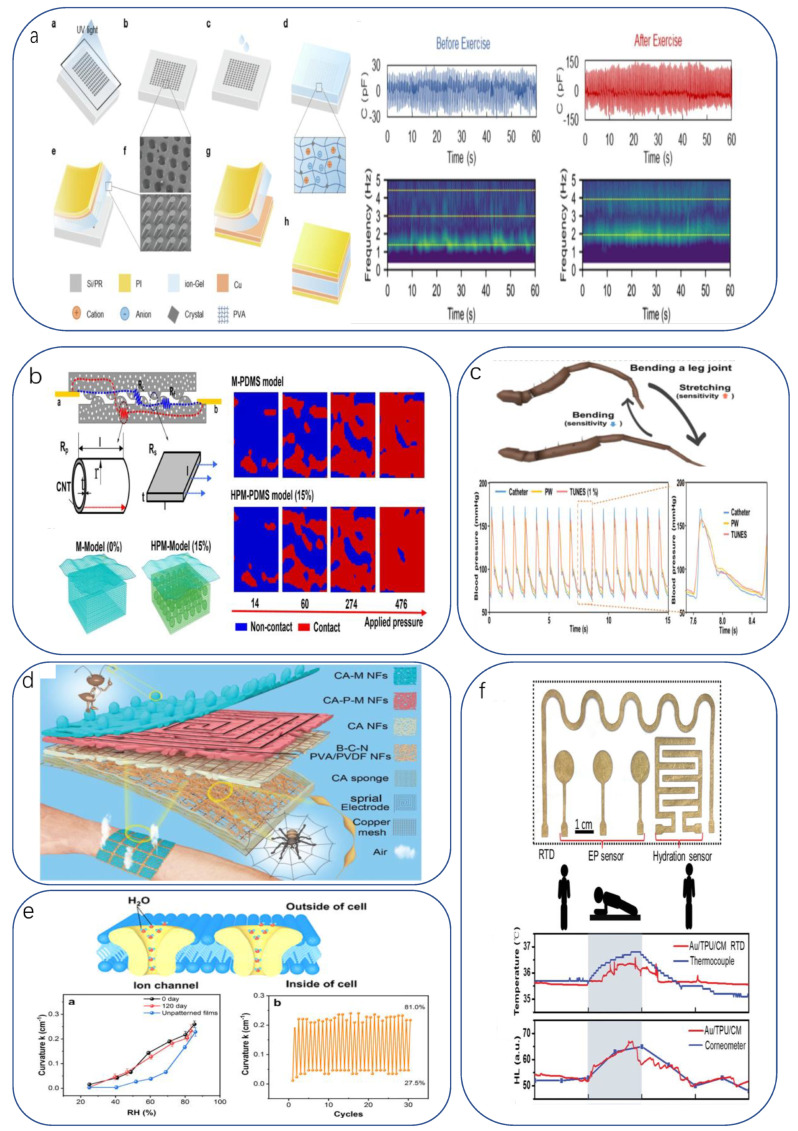
(**a**) Principle and sensing mechanism of hair-like microstructure design (Figure a~h left: Schematic process for the sensor fabrication; Right: Flexible sensors using for pulse wave monitoring without external pressure) [209]. Copyright 2022, Elsevier. (**b**) Working mechanism of HPM-based sensors [193]. Copyright 2019, American Chemical Society. (**c**) Schematic of sensitivity tuning mechanism of spiders; application in mice and clinical trials [216]. Copyright 2023, Springer Nature. (**d**) Structure, composition, biocompatibility, and permeability [217]. Copyright 2021, John Wiley and sons. (**e**) Schematic illustration of water molecule transport in biological ion channels and asymmetrically patterned CNF/GO composite membrane monitoring [220]. Copyright 2020, American Chemical Society. (**f**) Au/TPU/CM-based multimodal epidermal sensor [223]. Copyright 2022, John Wiley and sons.

Pulse waves are an indication of current cardiac and vascular performance and sympathetic nerve action in the heart and lungs. Monitoring pulse waves during surgery is an important reference for physicians to understand the physical status of their patients. Human skin itself is an excellent pressure sensor. The composition of the skin surface hairs and the subcutaneous mechanoreceptor pairs form an excellent pressure–electricity conversion mechanism. When pressure is applied, the angled hairs transmit the pressure to the skin [209]. Under the skin’s dermis, the perimeter of the hair ends is covered with low-threshold mechanoreceptors (LTMRs) [210]. Inspired by this mechanism, a hair-like microstructure was designed to improve sensitivity in the high-voltage interval. The sensor is a simple sandwich structure. Both the top and bottom electrodes consist of copper foil and a PI membrane. A special ionic hydrogel membrane contains an array of inclined cylindrical microstructures located in the middle. The wafer with photoresist is lithographed by light incidence at a tilted angle during the exposure process to obtain a wafer template with an unstable tilted microstructure. The hydrogel solution is combined with the template and dried to obtain an ionic hydrogel film with the desired microstructure. The sensor can easily perform pulse wave detection, obtain pulse waves from arteries at different locations, and detect heart rate changes. In combination with the different pressures of the obtained pulse wave signals, further calculations can determine the blood pressure (Figure 4a). Similarly, in Zhao’s study [193], a pressure sensor based on HPMs was designed and fabricated using sugar as a porous template and duplicating the surface of *Epipremnum aureum* leaves, demonstrating a potential application in wrist pulse detection. By imitating the unique layered surface of *E. aureum* leaves, the P-PDMS surface could have macroscale veins and microscopic surface microstructure. The produced pressure sensor exhibited a sensitivity of 83.9 kPa^−1^ (<140 Pa), a detection limit of 0.5 Pa, and good stability over more than 28,000 cycles of testing (Figure 4b). Electrocardiogram is an examination method that records the electrical activity of the heart. ECG can be used to quickly diagnose a considerable number of heart diseases, such as arrhythmia, myocardial ischemia, the site of myocardial infarction, and to determine the effect of drugs or electrolyte conditions on the heart, so it is widely used in clinical practice. Chun et al. developed a GCF (graphene-coated fabric) sensor that can also be attached to human skin by imitating the suction cup structure of an octopus [211]. This creates a strong adhesion to the skin surface (and this adhesion is equally effective in water). The sensor responds reliably to both pressure and strain, with a maximum pressure sensitivity of 0.74% kPa^−1^ (0–35 kPa), a minimum detected pressure of 12 Pa, and a minimum detected strain of 0.5%.

Spiders lower the threshold at which the sensory organs receive external compression/tension signals by changing the leg posture so that the sensitivity of the sensory organs becomes tunable [212,213], becoming insensitive in the closed state of the leg and sensitive in the extended state, with a 224-fold difference in sensitivity before and after closure [214]. This adaptation to the external environment adjusts the sensitivity, resulting in a wide range and improved detection [212,215] to the benefit of researchers. Recently, Taewi Kim developed and demonstrated tunable, ultrasensitive, nature-inspired epidermal sensors (TUNES) [216], which can dramatically adjust the sensitivity of pressure sensing of a signal by a preset strain. This tunability is due to the nonlinear characteristics of the nanofracture-based sensor. The study demonstrated that a single sensor could measure different biosignals, such as respiration, muscle contraction/relaxation, and minute wrist pulses, and was demonstrated in clinical trials and compared with commercial medical devices without losing ground (Figure 4c). In the future, it may be possible to classify the pulse signals of young and elderly groups.

Physiological activity includes not only movements of muscles and joints but also temperature changes and sweating. The detection of local temperature changes and dehydration in biological tissues is an issue in healthcare. Human skin is sensitive to pressure and temperature, but it lacks receptors that are sensitive to humidity, so it performs poorly regarding humidity perception. Researchers want to remedy this deficiency and make it easier to accurately analyze the environment. One work has shown that an ant’s tentacles can sense humidity, odor, sound, etc. [217]. Ouyang designed an “antenna” (Figure 4d) on the surface of CA-M NFs to mimic an ant’s tentacles as the top layer (humidity sensing layer) of the device [218]. The increased contact area between the water molecules and the NF-based structural layer allows the former to complete infiltration faster. This resulted in response and recovery times of 16 and 25 s, respectively. The humidity range was 25–85% RH. Other layers were spun with spider webs as inspiration to obtain CA-PEDOT-MWNTs NFs as temperature sensing layers. The ultrafast transport characteristics of bioion channels embedded in plasma membranes are essentially due to the gatekeeper-like function of selective proteins [219]. Inspired by bioion channels embedded in plasma membranes, Zixiu Li reported for the first time that asymmetrically patterned CNF/GO composite membranes prepared by vacuum-assisted filtration and surface printing techniques could be used as novel humidity sensors [220]. The periodically patterned channels on the surface of the hydrophilic CNF/GO composite membrane emulate specific selective proteins in biological ion channels, which bind to water molecules to induce a fast and orderly flow of water molecules in the form of single molecule passage, enabling orderly and ultra-fast water transport and thus improving its responsiveness to water stimulation (Figure 4e). Plants use the layered porous structure of catheters to achieve water transport from the soil to the stem and leaves [221]. Spider silk with tapered knots uses the radius difference of the fibers to guide the directional movement of water droplets [222]. Inspired by this liquid transport strategy, the team of Yanting Xu demonstrated a dermal gold/TPU/cellulose membrane (Au/TPU/CM) skin electrode with dual porosity and surface energy gradients [223], which conducts sweat unidirectionally from the sensor/skin interface to the sensor surface. Of particular note is its excellent moisture vapor transmission rate (2.2 times and 7.1 times that of cotton fabrics, respectively). The ultra-fast wicking ability improves the wearer’s comfort, which is important for the device: on the one hand, it minimizes temperature measurement errors due to skin hydration caused by sweating; on the other hand, it reduces the influence of noise caused by electrolytes and the risk of short-circuiting the sensor array due to external conductivity, thus significantly improving the precision and reliability of electronic skin sensing (Figure 4f).

#### 3.1.2. Treatment Aids

**Figure 5:**
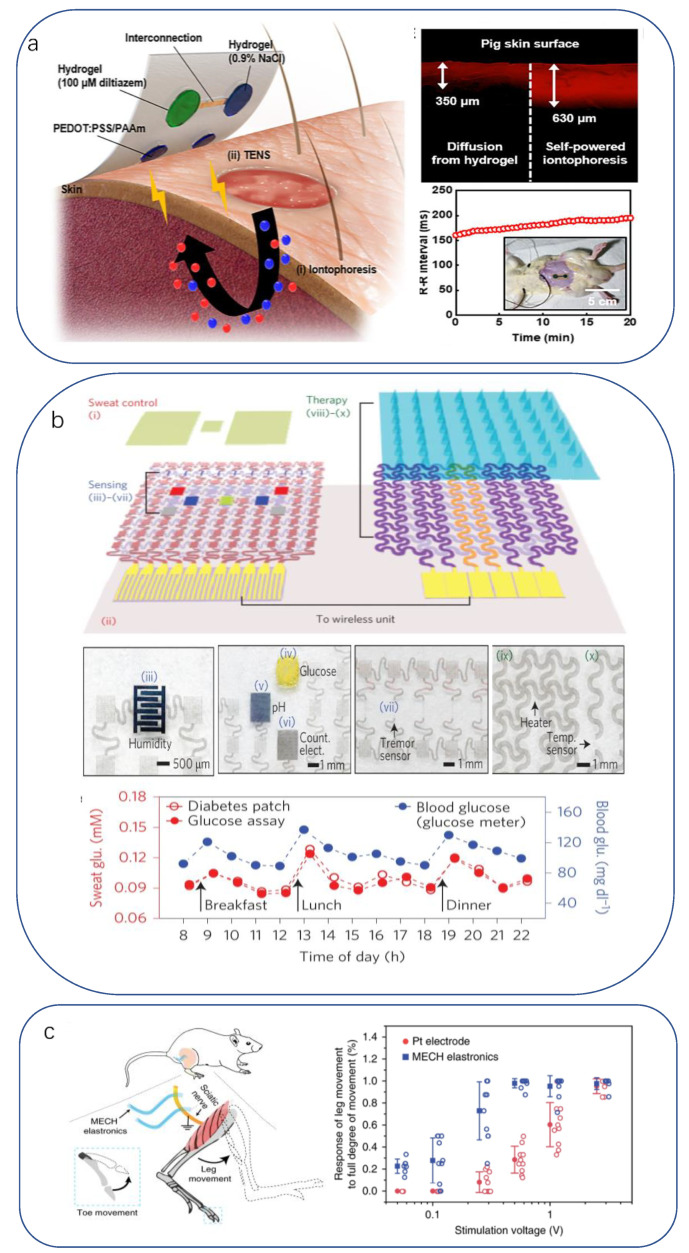
(**a**) Self-powered drug dialysis device with application of iontophoresis patch for in vitro drug delivery to pig and mouse skin [227]. Copyright 2021, American Association for the Advancement of Science. (**b**) Multiplexed sweat sensors monitoring temperature, humidity, glucose, and pH [4]. Copyright 2016, Springer Nature. (**c**) In vivo neurostimulation experiments using MECH microelectrodes [235]. Copyright 2019, Springer Nature.

The analysis and treatment of open wounds (transdermal), such as local oxygen concentration measurement [224], impedance sensing [225], drug release [226], and electrical stimulation, are widely used in medical activities for bruises, postoperative treatment, and previous wearable devices and are usually accompanied by bulky electrochemical sensing devices or drug delivery systems, which limits their application in personalized medicine. New light and soft skin-like bionic sensors mimic biological self-protection for wound healing and self-regulation of the physiological environment. In this application, in addition to the basic hygienic requirements of moisturization, osmotic pressure, and barrier to germs, uniform drug delivery rates are achieved with the timely assessment of the physiological environment of the wound. Lim Chanhyuk’s team proposed a skin–device interface with biological tissue characteristics using an ultra-thin polyacrylamide (PAAm)-based hydrogel [227]. It remains in close contact with the undulating skin surface due to its ultra-soft mechanical nature. Its porous three-dimensional network structure and ultra-thin thickness allow for rapid diffusion and transport of target bioanalytes and drug molecules in both forward and reverse directions. Using this high-quality, highly permeable, and low-impedance ultrathin hydrogel sensor, a high-performance iontophoretic drug release device (Figure 5a) was developed for transdermal bioanalysis, therapeutic, and pharmacological clinical trials [155]. Kim integrated a heater as well as temperature, humidity, glucose, and pH sensors into a device that could be applied to the skin [4,228] (Figure 5b). Once sweat secretion reaches a critical humidity level, the three sensors above operate simultaneously, and the other two can be appropriately corrected for glucose readings to improve accuracy. The device also integrates microneedles that activate a heater when the glucose sensor detects a high glucose concentration, and the microneedles release antidiabetic drugs when heated. Another example is the artificial skin coating that “sweats” when stimulated by radio waves, generating droplets of ibuprofen or other drugs that both keep themselves clean and deliver drugs to wounds [229]. 

The neural system is the center of the human body, and all physiological activities and sensations are regulated and transmitted by the neural system. Behavior and response to external stimuli are controlled by the repeated alternation of electrical and chemical signals between neurons. Medical research and treatment options for neurological disorders are important topics in the field of medical health. In the past few decades, several bio-inspired flexible electronic devices have been developed, including fibrous nerve electrodes that mimic the structure of neurons and their processes [230,231] and networked neuroelectronic devices that mimic the structure of neural networks in the brain [232,233]. Bio-inspired fibrous electrodes with cellular/subcellular size and mechanical strength can significantly reduce tissue damage during implantation, thereby reducing inflammatory responses [234]. Yu Xin et al. [235] described a method for fabricating highly conductive neural electrodes that tunes Young’s modulus of active and insulating materials to match that of the nerve tissue. Multi-electrode arrays manufactured by a lithographic process with good hydrostability and biocompatibility were chronically implanted into the bodies of mice to produce effective electrical stimulation and high current density at ultra-low voltage (Figure 5c). The same work was conducted with Neurotassel [236], a high-density filament-mimetic neural probe developed by Li’s group with a volume approaching the size of a single neuron. This probe has a unique planar reticulate filament structure designed for small size and flexibility, resulting in minimal loss of neurons around the implantation site, and the results show stable tracking of 6 weeks of activity in the mouse medial prefrontal cortex.

**Figure 6:**
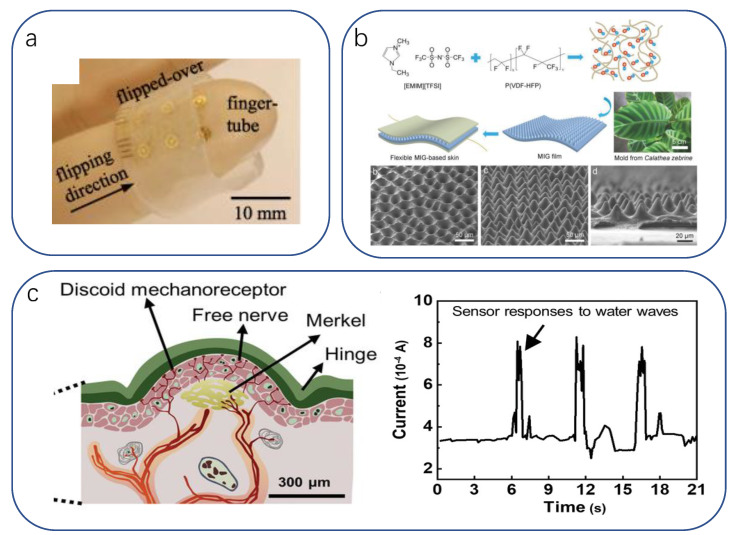
(**a**) Optical photograph of the fingertip of the prepared surgical glove [236]. Copyright 2012, IOP Publishing. (**b**) Schematic of the fabrication of a mechanically flexible capacitive sensor with a MIG dielectric layer [191]. Copyright 2018, John Wiley and sons. (**c**) Left: Schematic of the pressure-sensing organ of the crocodile. Right: change in current of the pressure sensor attached to the crocodile’s jaw in response to water waves [246]. Copyright 2022, John Wiley and sons.

The involvement of medical robots/hands in medical activities and how they can match or even exceed the behavioral level of human surgeons in such a challenging activity as surgery has attracted numerous studies. Earlier electrode arrays of silicon nanomembranes were integrated into closed tube geometries applied to the fingertips, enabling surgical gloves to achieve electrotactile stimulation mimicking the physician’s hand touch [236] (Figure 6a). Combining large-area thermal and pressure sensing will further refine its humanoid function [11]. Electrostimulation can be used to control balance in patients suffering from vestibular disorders [237,238,239,240,241] and compensate for damaged neural function. Sophisticated medical operations demand high sensitivity in medical device receivers. Qiu [191] et al. fabricated a flexible capacitive pressure sensor using Calathea zebrine leaves as a template. The basic structure is an ionic gel film with the same microstructure as the bamboo leaf, which is fabricated in the center of the electrode layer using a two-casting method. The sensor has a sensitivity of 54.3 kPa^−1^ over a pressure range of 0.5 kPa and a minimum detection pressure of 0.1 Pa. The response and recovery times are 29 and 37 ms, respectively. The sensor is still functional under 5400 cycles (Figure 6b), which improves the sensitivity of human–computer sensing. The skin of crocodilia has highly sensitive dome-shaped pressure-sensing organs with hinged structures, and they enable crocodilia to sensitively detect mechanical stimuli [242,243] and even tiny ripples on the water surface [244,245]. Such a highly sensitive working mechanism is undoubtedly a great advance in the haptic perception of smart medical devices to ensure that the risk of harm to humans from the devices is minimized. To this end, Jiyuan Li developed the first method to fabricate an omnidirectional stretchable pressure sensor [246]. The high sensitivity of this stretchable pressure sensor is achieved by selectively depositing long and short silver nanowires and using surface folds to maintain the resistance of the electrodes. Thus, even at 100% uniaxial strain or 50% biaxial strain, only slight changes occur. However, this work has not yet been applied to rigorous medical experiments (Figure 6c).

#### 3.1.3. Clinical Studies

**Figure 7:**
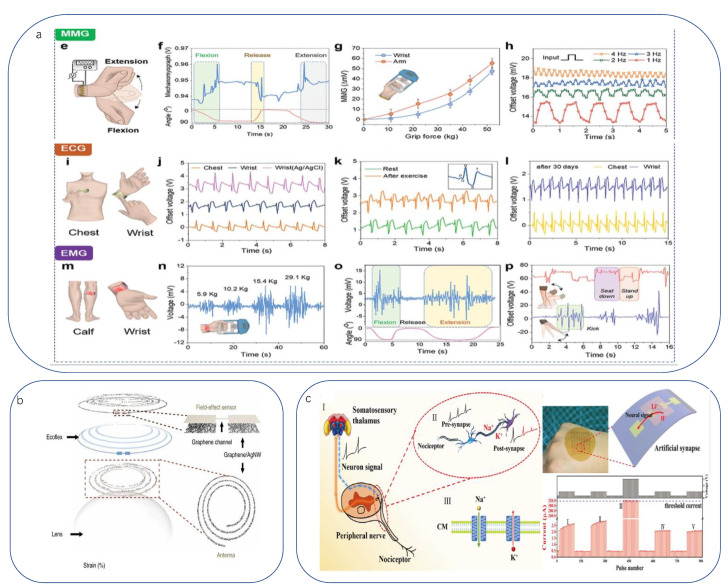
(**a**) Multimodal skin sensors monitoring physical and electrical signals on the wrist, chest, and leg. From left to right—MMG: MMG sensor attached to wrist and expressions for flexion and extension; MMG signal according to wrist angle; Signal values from MMG sensor attached to the arm and wrist according to grip force; Voltage generation of MMG sensor according to frequency changes. ECG: ECG sensors attached to chest and wrist; Signal obtained by ECG sensor. Comparison with commercial ECG (Ag/AgCl); Comparison of ECG signals at rest and after exercise (light walking). (Inset: typical ECG signal and main peaks); ECG measurements for sensor stability after 30 days. EMG:EMG sensors attached to calf and wrist; EMG signal measured while increasing gripper force; EMG signal measured while changing wrist angle; Signal acquisition according to activity type of EMG sensor attached to calf. (Top: standing and sitting down, bottom: kicking) [247]. Copyright 2022, John Wiley and sons. (**b**) Intraocular sensors fabricated on soft contact lenses with antennas for wireless communication [248]. Copyright 2017, Springer Nature. (**c**) Schematic diagram of pain perception mechanisms and studies of biological. Lower right corner: Response of the post-synaptic current to mild stimuli. The dash line shows the threshold current for the functional loss of the nerve system [249]. Copyright 2020, Springer Nature.

Today there are still many diseases, and in the development of human health law, there are still no definite unified research conclusions, so clinical research is an important issue in the medical field, meaning sensors in this field should also play a full role. Qin’s team developed an artificial, wearable, all-gel multimodal skin sensor [247] that is flexible and easy to assemble; multiple biophysical signals (blood pressure, electromyography) can be monitored simultaneously on the wrist. The selected PANi-polyvinyl chloride (PVC) ionic composite gel material was found to be well suited for detecting slow-response characteristics such as blood pressure (to be discussed later), while polyvinylidene fluoride-trifluoroethylene (PVDF-TrFe) gels are more suitable for detecting fast-response characteristics such as electromyography. Researchers evaluated the relationship between cardiovascular health and muscle exercise by concurrently monitoring the correlation between muscle kinematics and hemodynamics (Figure 7a). In addition, Park fabricated a translucent, stretchable multifunctional sensor on a soft contact lens using a sandwich structure, a deformable dielectric layer (Ecoflex) sandwiched between two sensing helical electrodes (graphene/Ag NW). This sensor can be placed on the eye in a cornea-like structure to detect IOP (used to monitor the pathogenesis of glaucoma) wirelessly [248] (Figure 7b).

In addition to studying the pathogenesis of diseases, quantifying pain helps to communicate empathy between the physician and the patient. Pain is a biological self-protection response. On the one hand, it can cause avoidance behavior by sending nociceptive signals to the hypothalamus to protect against further injury. On the other hand, it can prevent the overexpression of neuronal signals in the event of neuropathic damage caused by painful stimuli. Differences in patient tolerance in real-world medical treatment mean that the determination of a patient’s level of pain relies on doctor–patient communication, with no quantitative indicators. A great deal of work has been carried out to comprehend the mechanisms of pain. Many studies have shown that ion channels in neurosynapses are key in determining adaptive nociceptive mechanisms. Inspired by the working mechanism of the biological nociceptive system, Lu designed a memory-based artificial synapse with a bilayer of active materials [249]. The material chosen for the top layer was LiClO4-doped polyoxyethylene (PEO:LiClO4), a polymer with bioadhesive, mucoadhesive, and biocompatible properties. Proton and Li+ mimic the presence of Na+ and K+ in biological synapses, respectively, and when Li+ is inserted into s-SWCNTs, its electrical characteristics can be easily tuned due to its small atomic size. As a substrate material, 90% of semiconducting single-walled carbon nanotubes (s-SWCNTs) have been selected. The device successfully simulates biological nociception and nerve damage by exhibiting synaptic-like operating potentials in the low-voltage range and inhibitory effects in the high-voltage range. We can provide convenience for medical research by seeking evidence and explanation of the principle of the working mechanism of subsynaptic nociception (Figure 7c).

### 3.2. Application of Bionic Flexible Sensors in Rehabilitation Assistance

Usually, the patient’s recovery presents two extremes, one is purely dependent on the patient’s own physical quality to overcome the post-sickness/post-operative discomfort, and the medical treatment can only be passively supported by medication, lacking timeliness, so the patient’s pain relief needs to be improved; the other is a serious illness/major surgery causing great damage, meaning patients cannot leave the continuous companionship of doctors and medical devices, exacerbating the burden on medical resources. Biologically inspired devices are able to compromise between the two scenarios by taking advantage of their inherent flexibility, low cost, high sensitivity, and biocompatibility. This can be achieved by reducing the complexity and cost of the device by using natural examples in the design of the device while achieving specific requirements other than functionality, improving the reliability and sensitivity of the device in terms of structure and function, and improving the mental anguish and physiological incongruity caused by a lifeless device.

**Figure 8:**
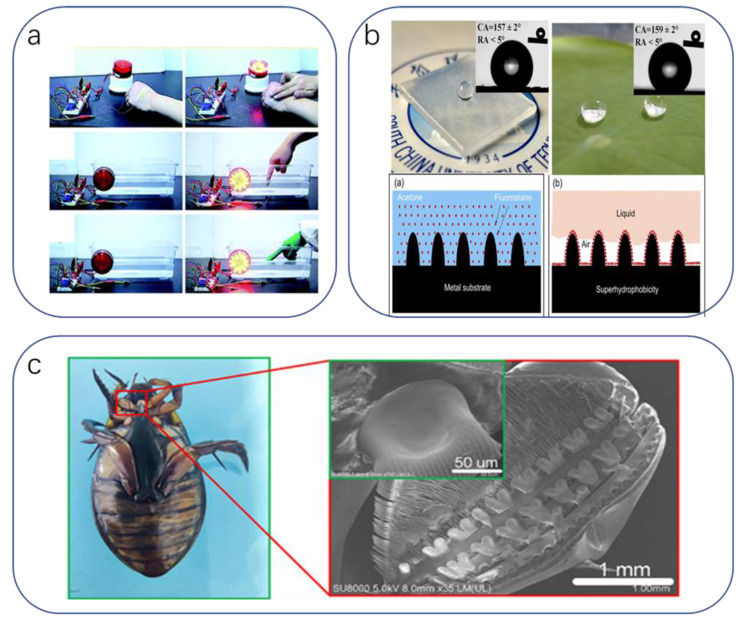
(**a**) Application of MIS-based sensors for real-time detection of motion and vibration in an aqueous environment [250]. Copyright 2021, Royal Society of Chemistry. (**b**) Schematic of the superhydrophobic surface of pp replicas and lotus leaves (Insets show wetting state of 4-μL water droplets), pictured below is the fabrication of the nanotextured layer [251]. Copyright 2019, Elsevier. (**c**) Photographs of the top and bottom features of *D. lapponicus* and SEM images of the setae in its attachment pad [254]. Copyright 2021, American Chemical Society.

Swimming is useful for a variety of diseases (e.g., fractures, arthritis, neurological disorders, etc.) and for post-operative recovery, improving body coordination and strengthening the respiratory, neurological, and digestive systems with the protection, support, and thermoregulation of water buoyancy. While many conventional in-water monitoring devices make it difficult for physicians to track the patient’s swimming training process due to circuit characteristics, size, and wiring issues, biologically inspired devices reduce the requirement for the physical fit of components and avoid the use of chemicals such as sealants, which reduces the requirements and cost of the manufacturing process, while reducing the size of the sensor by simplifying the required device structure. As the field of healthcare rehabilitation evolves toward individual patient monitoring, thin, flexible, and water-resistant bionic flexible sensors are becoming an urgent need for physicians in swimming rehabilitation. For animals in nature, the scale structure of the skin surface, which is isolated from the internal and external environment, provides good hydrophobic performance. Ma reported a highly elastic, durable, and ultra-sensitive micro-nanostructure-based skin-like ionic gel (MIS) [250] with good sensing performance to various stimuli (e.g., strain, pressure, and temperature). The MIS-based wearable sensor is used for real-time monitoring of various human motions and is particularly characterized by its excellent superhydrophobicity, which makes the sensor promising for use in harsh environments with high humidity and even underwater (Figure 8a). Meanwhile, we can take the hydrophobic examples of natural plants (lotus leaf [251], purple orchid leaf [252], etc.) as inspiration. For example, Huang used a natural lotus leaf as a natural template and made a nickel template by electroless plating and electroplating to inject a lotus leaf-like structure on the surface of polypropylene (PP). The results showed that the nano-microvilli with sharp tips and high aspect ratio on the lotus leaf were successfully replicated on PP replica [251] without any additional surface coating or chemical modification, so it is an industrially feasible method for large-scale fabrication. The PP replica exhibits very similar wettability to that of the lotus leaf. It even surpasses the natural performance of the lotus leaf in terms of dynamic superhydrophobic stability and superhydrophobic thermal durability (Figure 8b). Guo used a laser direct-written bionic rose petal microbubble structure and achieved superhydrophobicity by coating the surface with fluorosilane [253]. This microbubble structure perpendicular to the substrate effectively enhanced its surface hydrophobicity to liquids; the surface consistently exhibited excellent superhydrophobic properties in both underwater immersion, bouncing behavior, and droplet rolling tests. Not to be overlooked is the fact that water scouring from the swimming motion challenges the strength of the device. The Dytiscus lapponicus-inspired structure [254], which has high adhesion in dry and underwater environments, was developed to take advantage of the fact that the sticky setae on the ventral side of the front legs of Dytiscus lapponicus males firmly attach to the rough shell of females during mating. The bionic structure of the sticky setae ensures good adhesion to the skin surface in both dry and wet conditions (Figure 8c).

**Figure 9:**
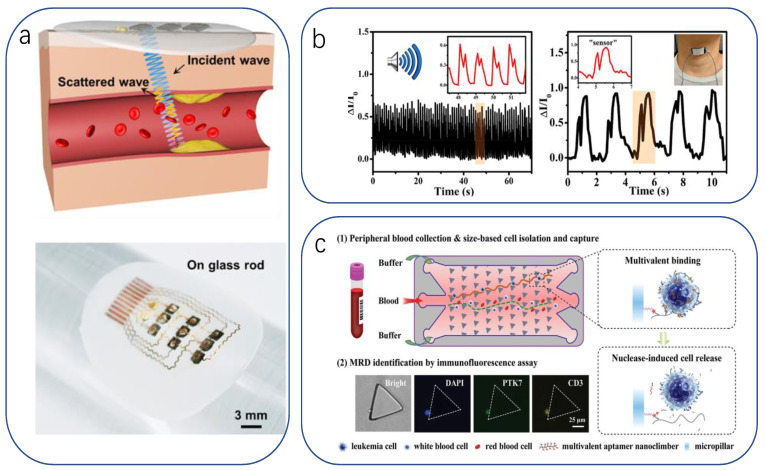
(**a**) Above: Schematic diagram of the ultrasound device. Receiving echoes from a moving scatterer (e.g., red blood cells). Lower: Optical image of the device as it bends around a curved surface [255]. Copyright 2021, The American Association for the Advancement of Science. (**b**) Small vibrations induced by music (cyclic drumming) and recognition of speech [256]. Copyright 2019, American Chemical Society. (**c**) Schematic diagram of the MANC-Chip capturing and releasing leukemic cells in peripheral blood [261]. Copyright 2020, John Wiley and sons.

After revascularization surgery, there are sequelae such as thrombosis and restenosis that can be severe enough to cause stroke, but there is a lack of clinical means to continuously monitor vascular conditions. Conventional probes are based on the principles of photoelectric or thermal analysis, resulting in the measurement of only relative values of changes in vascular flow. Utilization of the high-performance bionic ultrasound flexible sensor can support non-invasive, real-time, and continuous monitoring of blood flow velocity in human arteries, with the critical selection of material composition and microfabrication optimized processing procedures ensuring that it provides satisfactory signal quality despite the absence of coupling agents [255] (Figure 9a). Zhou et al. fabricated a piezoresistive pressure sensor with a bionic inner–outer epidermal structure [256]. This was achieved by assembling a PDMS film face-to-face with a carbon powder/polydimethylsiloxane (CPDMS) conductive composite film with a microdome structure. Based on tests, this resistive pressure sensor has a sensitivity of 124 kPa^−1^ (0–200 Pa), with other high pressure ranges dropping from 0.39 kPa^−1^ (0.2–12 kPa) to 0.02 kPa^−1^ (12–50 kPa); the human acoustic pressure range only included the most sensitive range, with a detection limit of 2 Pa. The response time was about 100 ms (Figure 9b), which can be used for rehabilitation analysis of sound state frequencies after vocal fold surgery. Weng fabricated a novel rGO pressure transducer [257]. rGO films have a bionic crack structure that can be formed by simple stretching and release. With a sensitivity of up to 5.77 kPa^−1^ (0–490 Pa) and 0.25 kPa^−1^ (490–9800 Pa), as well as a fast response time of 97 ms and recovery time of 98 ms, the researchers demonstrated that although it is less sensitive than the previous device, it can still create applications by combining it with other amplification devices. The researchers demonstrated that by integrating a pressure sensor into a translation system using Moss code encoding, the pressure sensor taps into the translation system with the initial expression as an electrical signal in the form of a combination of short dots, short lines, and long lines. This work could help people with amyotrophic lateral sclerosis express their thoughts and needs to family, friends, and therapists. Finally, Jian fabricated a flexible piezoresistive sensor with a biomimetic plant surface microstructure [258]. Using the golden leaf as a mold, he prepared a PDMS film on the leaf’s microstructured surface and an ACNT/G film on the other side, which was then transferred to the PDMS film. Experiments were conducted to attach the ACNT/G pressure sensor to a loudspeaker to verify its ability to detect and discriminate different sounds. The peak and trough values of its response curve for the same word were found to have very similar characteristics when compared several times, indicating the high sensitivity and excellent reliability of the sensor for speech recognition.

Minimal residual disease (MRD) refers specifically to the small number of leukemia cells that remain in the patient’s body during or after treatment. Its monitoring can be used to assess the patient’s response during treatment, to help determine the patient’s subsequent risk-adapted treatment regimen, and to monitor the burden of stem cell transplantation postoperatively [259,260]. The bone marrow aspiration sampling method currently in clinical use is highly invasive and causes predictable physical and psychological pain to the patient, making frequent sampling impossible [261]. Therefore, there is an urgent need to develop a less invasive and clinically acceptable new technique for MRD testing. Inspired by the adaptive skeleton and multiple suckers or tendrils of climbing plants, Liu’s team reported a biomimetic multivalent ensemble nanochip (MANC)-functional microfluidic chip for in vitro MRD detection in patients with T-cell acute lymphoblastic leukemia [262]. Upon collection of a blood sample, a rotating triangular micropillar in the MANC chip divides it into multiple streamlines. Corresponding streamlines with leukemia cells larger than the critical diameter are transferred and captured (Figure 9c).

**Figure 10:**
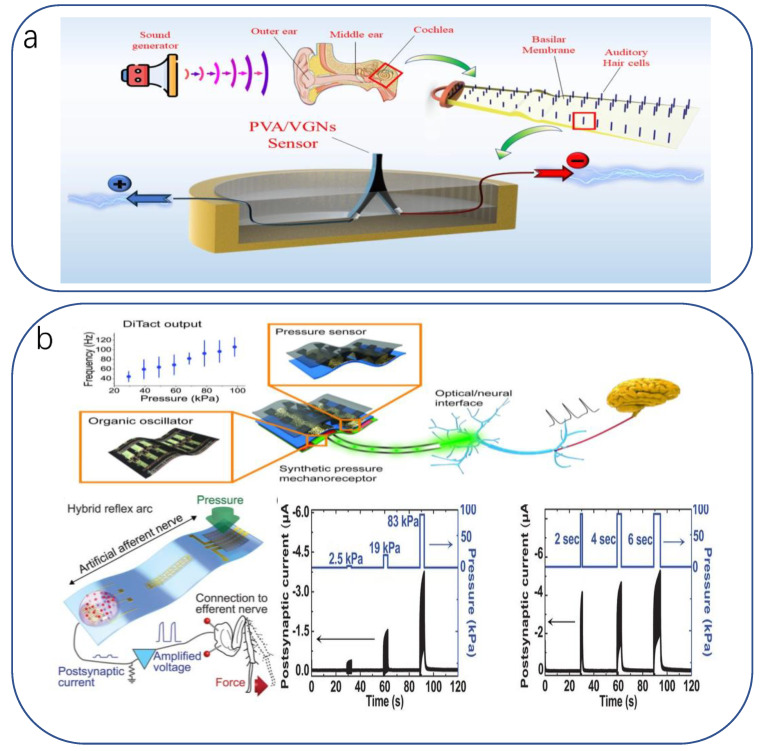
(**a**) Ultra-sensitive bionic auditory hair cells based on piezoresistive hydrogel nanocomposites [263]. Copyright 2021, American Chemical Society. (**b**) Schematic diagram of tactile sensor signal processing using artificial biological structures (e.g., synapses, mechanoreceptors, or both) [49] and flexible organic artificial afferent nerves [265]. Copyright 2019, John Wiley and sons. Copyright 2018, The American Association for the Advancement of Science.

The cochlea is a very sensitive and complex sensory end organ, and the cochlear hair cells are its specialized mechanoreceptors, which are the basis for mechano-electrical transduction and sensory coding in bionic work. The cochlear implant receives sound vibration signals from the environment, which are processed and converted into electrical stimuli to act on cochlear neurons, helping to restore hearing in patients with impaired or dysfunctional cochlear hair cells. Ahmadi demonstrated the infiltration of PVA hydrogels into vertical graphene nanosheets (VGNs) to develop a piezoresistive hydrogel-based acoustic sensor that mimics the behavior of auditory hair cells in the cochlea [263] (Figure 10a). The flexible polymer PVA was used as a substrate for the growth of the VGN network film on a copper substrate. This PVA/VGN piezoresistive transducer can perform normal acousto-electric signal conversion for sound vibrations ranging from 60 Hz to 20 kHz.

Cochlear “prostheses” have no neurological interaction and are designed more like conventional wearables. However, in most cases, there are two clinical problems in the rehabilitation and adaptation of human prostheses: one is the phantom pain of the newly amputated patient, which can be alleviated over time with analgesic medication and psychological counseling, and the other is the lack of neural interaction between the prosthesis and the organism, which can take a very long time to adapt to the user and create dependence on the adapted prosthesis. To address these issues, researchers have begun to try to provide the organism with more signals about the prosthesis; in particular, how signals from the prosthesis receptors are transmitted to the organism in the form of human neurotransmission. Han implemented an artificial sensory neuron by connecting a piezoresistive pressure sensor that emits spike signals to a naphthalene-based memristor [264]. The results showed that the cellular potentials measured in the brain slices behaved very similarly to the spike signals generated by the sensor. This indicated that the artificially generated spike signals might be related to the real human nervous system [49]. Lee proposed a simulated afferent nerve system based on the notion of artificial mechanoreceptors and neurosynapses (Figure 10b), and the researchers connected the artificial afferent nerve to the biological efferent nerve of the discoid cockroach to mimic a biological reflex arc [265]. Thakor quantified the difference between tactile and pain signals by analyzing the resistance changes in a multi-layered electronic skin consisting of multiple tactile sensors in the dermis/epidermis, and the amputees were able to perceive the corresponding stimulation and pain when the spike signals were transmitted to the upper extremity through the stimulator.

### 3.3. Application of Bionic Flexible Sensors in Health Prevention

There are two realistic factors at play in health prevention. One is that, compared to the previous two categories (diagnostic treatment and rehabilitation), where patients can make compromises for healing, users in this category are more concerned about the comfort and cost of the device while taking into account the accuracy of monitoring. The other is that the real environment has a variety of complex factors that can affect the human body, yet the current development is mainly focused on common human indicators (blood pressure, blood oxygen, blood glucose, etc., which are often the main areas of commercial development), but in other indicators of the human body, especially hormones, enzymes as representatives of the endoplasmic indicators of the lack of development, while convenient and rapid monitoring means for environmental factors are still rare, which makes the current sensor monitoring the field is relatively small. In contrast, real creatures have improved in structure and function over millions of years of development: some have made their structure more comfortable and cost-effective, some have developed specific responses to certain substances to avoid harm, and some are particularly sensitive to changes in environmental factors. This has allowed us to use them as inspiration for both conventional monitoring areas and the development of more targeted devices to expand the scope of monitoring.

#### 3.3.1. Health Status Monitoring

**Figure 11:**
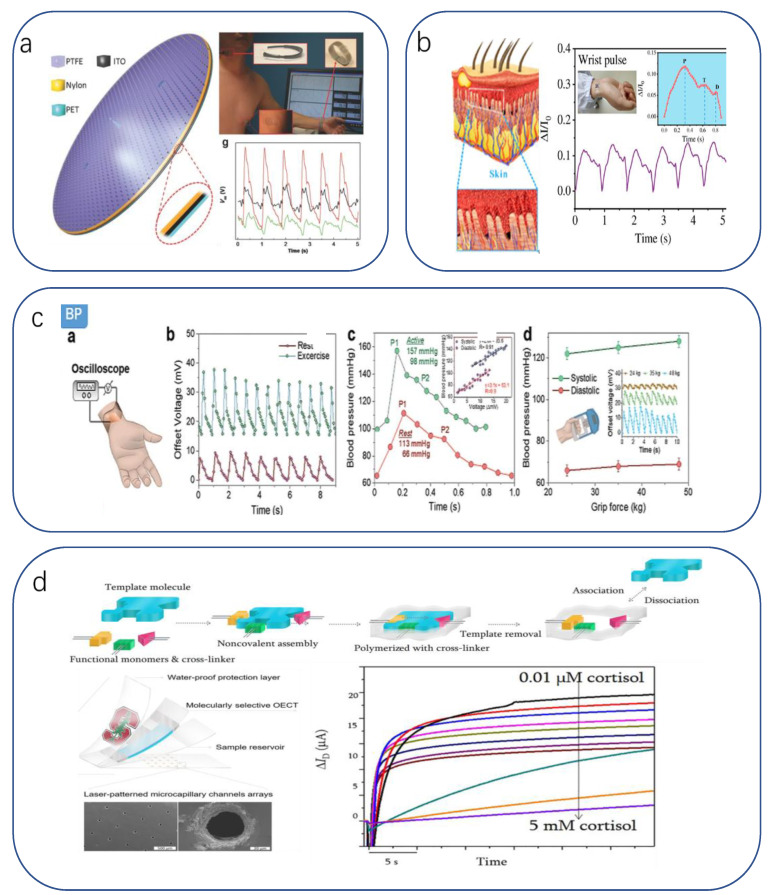
(**a**) Schematic of an eardrum-inspired self-powered sensor [268]. Copyright 2015, John Wiley and sons. (**b**) Human skin and subcutaneous spike-like microstructure and monitored wrist pulse (enlarged image is a magnified view of the pulse vibration waveform) [269]. Copyright 2020, American Chemical Society. (**c**) Wrist-mounted blood pressure sensor measured with an oscilloscope [247]. Copyright 2022, John Wiley and sons. (**d**) Top: Schematic diagram of the recognition site obtained by copolymerization of functional monomers and cross-linkers in the presence of analytes with binding affinity close to that shown by the antibody-antigen system. Bottom left: Schematic diagram of a bionic patch-type cortisol sensor. Bottom right: Output current effect of spraying artificial sweat with different concentrations of cortisol [271]. Copyright 2018, The American Association for the Advancement of Science.

Today, early diagnosis and long-term monitoring of physiological signals can prevent most cardiovascular diseases, including atherosclerosis [266,267], myocardial infarction, and hypertension. Yang et al. first reported a flexible and lightweight tympanic-inspired bionic membrane sensor [268]. The device exhibited outstanding sensitivity of 51 mV Pa^−1^ based on contact electrification effects, a detection limit of 2.5 Pa, and a fast response time of less than 6 ms (Figure 11a). Impulse signals from the carotid, radial, and thoracic arteries can be measured and sensed at a low cost. The spiky microstructure of human skin allows for a high concentration of local stress in the contact area, inspiring us to improve the sensitivity and detection accuracy of the pressure device. A randomly distributed spike-like microstructure of a highly sensitive MXene-based piezoresistive sensor [269] was fabricated by thermal spraying using sandpaper stencil printing, where highly conductive monolayer Ti_3_C_2_TxMXene nanosheets were uniformly deposited with microstructured polydimethylsiloxane (PDMS) by van der Waals forces. As the pressure increases, the contact area of the bio-inspired microspines increases, essentially increasing the surface area of the conductive channels (lowering the impedance), thus improving the sensing performance of the pressure sensor (Figure 11b). The aforementioned multimodal skin sensor [247], using PANi-polyvinyl chloride (PVC) ionic composite gel as a key material, can be used to detect physiological signals with slow adaptive properties, such as blood pressure (Figure 11c).

A great deal of research in modern medicine has shown that emotions can have a direct effect on a variety of physiological activities in the body. Prolonged stress can disrupt the balance of the cardiovascular, immunological, and endocrine systems [270]. Under stress, the adrenal glands release cortisol and epinephrine into the bloodstream, which can be easily collected when the cortisol in the blood enters the sweat. Unlike the physical sensing approach, which is susceptible to environmental and other factors in the body, the extraction of the corresponding chemicals is more targeted. Onur Parlak et al. demonstrated the integration of an artificial receptor as a bionic polymer membrane [271]. A selective artificial receptor membrane based on molecularly imprinted polymers is placed between the PEDOT:PSS channel layer and the target analyte and serves to control the molecular transport of cortisol from the skin to the OECT sensing channel (Figure 11d). In these applications, the smaller size and lipophilicity of cortisol are exploited, and its diffusion in the sensor channel layer is inspired by its diffusion mechanism from blood to sweat. Coupled with microfluidics, results of the same quality as a large number of solution analyses can be obtained with a very small sample volume on the sensor surface. Further development of cortisol extraction and sensing was followed by validation of an attempt to use a flexible FET layer as a bionic polymer membrane, as described above [272].

#### 3.3.2. Motion Monitoring

**Figure 12:**
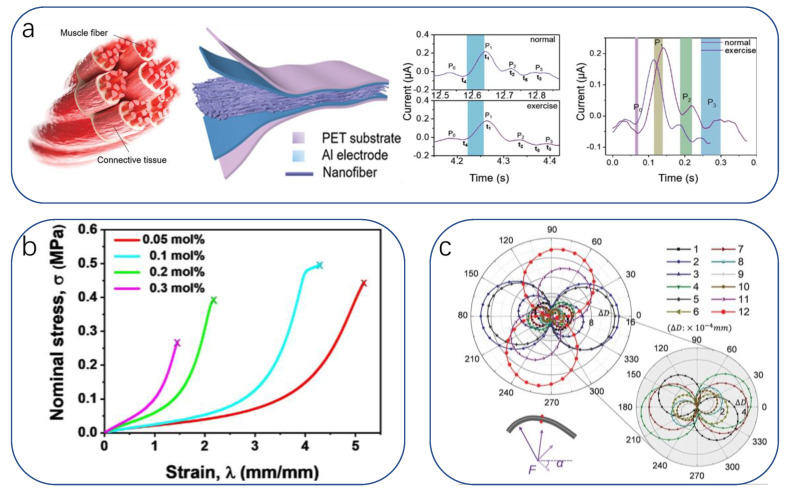
(**a**) From left to right: schematic of human muscle fibers; schematic of muscle fiber-inspired piezoelectric transducer; detailed analysis of pulse waveforms under normal and motion conditions, including three characteristic points (P1, P2, and P3) [274]. Copyright 2021, John Wiley and sons. (**b**) Tensile stress–strain curves of SMRH with different PAAm cross-linking ratios [275]. Copyright 2022, American Chemical Society. (**c**) Steering response of a pretreated scorpion, where one slit feeler remains intact, and the others are sealed up in response to target vibrations from different directions [276]. Copyright 2022, John Wiley and sons.

The connective tissue encapsulates the helical bundle of muscle fibers, enabling external load transfer and uniform stress distribution [273]. This unique soft biological structure significantly improves the mechanical strength and toughness of muscle tissue. By dispersing polydopamine in electrospun barium titanate/polyvinylidene fluoride nanofibers, Su et al. developed muscle fiber-inspired piezoelectric sensors with improved mechanical strength and interfacial adhesion [274]. The durability of the device was greatly improved (less than 3% degradation after 7400 operations), and human motion detection and pulse wave measurement showed high feasibility (Figure 12a). To more closely resemble the elastic mechanical performance of muscles, biological tissues are initially soft, and at certain values of tensile strain, their Young’s modulus increases by several orders of magnitude, exhibiting a nonlinear mechanical behavior that is of great value for device protection and for the skin patch. Recently, it was proposed that a biomimetic muscle hydrogel with both tissue-like mechanical properties and sensing capabilities was successfully fabricated by introducing polyelectrolyte PAAS microgels into the PAAm network [275]. It exhibited low Young’s modulus (22.61–112.45 kPa) and high tensile strength. The good strain–stiffness transition properties it exhibits are due to the entangled micro-regions of PAAS microgels and PAAm chains that can transfer stress to other chains. SMRH has great potential as a flexible strain sensor for monitoring physiological signals under human motion for practical wearable and implantable applications (Figure 12b).

Scorpions have evolved a powerful vibrating sensory organ called the slit receptor in their walking legs. It differs structurally from spider slit receptors due to habitat and environmental differences [276]. Several early biobehavioral studies have shown that ultrasensitive and omnidirectional sensing is a key survival tool for scorpions [277,278,279]. A bioinspired flexible strain sensor designed by Liu et al. revealed that visually degraded scorpions use fan-grooved slit receptors to detect subtle vibrations omnidirectionally [276]. The key to the sensing mechanism consists of curved microgrooves arranged around a central circle. Based on the nearly identical morphology of the slit receptors in the different legs of the scorpion and a very specific phenomenon: if only one slit receptor is fully functional and the others are closed, the scorpion still exhibits omnidirectional sensitivity. The researchers then developed a finite element analysis (FEA) model of the sensor dynamics as a function of stimulus orientation (Figure 12c), which confirmed the role of the slit sensilla’s bending properties and showed an unprecedented measurement factor of over 18,000 and stability of over 7000 cycles. This sensor shows a high degree of versatility as it can monitor a wide range of human vibratory movements, including the contraction–relaxation of forearm muscles, regardless of the sensor mounting angle, because of its ability to discriminate well between vibrations of different directions.

**Figure 13:**
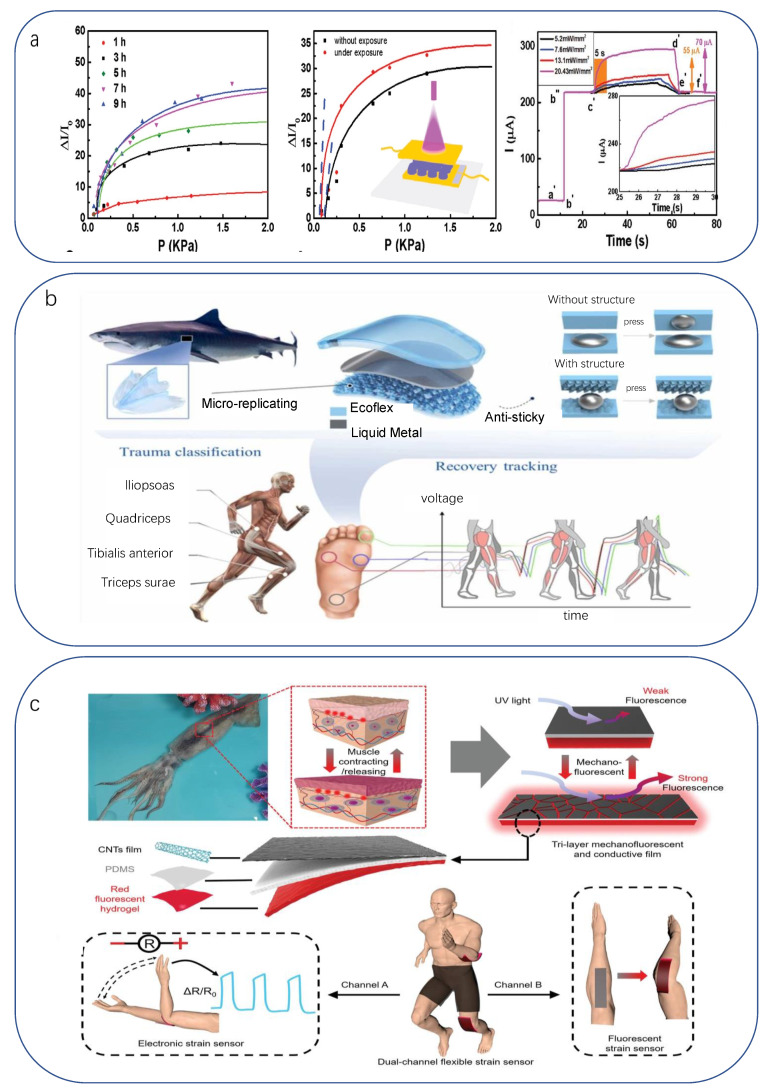
(**a**) Effect of illumination on the sensitivity of the pressure sensor (inset: schematic of the pressure sensor in the light condition), point a′, no pressure loaded; point b′/b″, starting loading the pressure; point c′ and point d′, turning on/off the light illumination, respectively; point b″ → point f′: the pressure loaded [280]. Copyright 2020, John Wiley and sons. (**b**) Bio-inspired sharkskin-imaged liquid metal frictional electrical nanogenerator for self-powered gait analysis and long-term rehabilitation monitoring [283]. Copyright 2022, Elsevier. (**c**) Mechanofluorescent and conductive hydrogel layer-based design of a bio-inspired dual-channel flexible strain sensor [284]. Copyright 2022, John Wiley and sons.

For teams of athletes engaged in competitive sports, individual-centered sensor monitoring has always been a top priority, i.e., this is responsible for the health of the athlete and the inevitable choice for chasing performance. From detecting body heat as an energy source to an electronic skin system with integrated multisensory receptors, multisensory functions include sensing temperature/humidity, wind and motion, and monitoring acceleration [186]. Yu used rose petals and a double casting method to prepare a rose surface microstructure of polypyrrole (PPy)/PDMS film [280], with a maximum sensitivity of 70 kPa^−1^ (*p* < 0.5 kPa.) The special feature that distinguishes it from other sensors is that light exposure can further increase the sensitivity (70 → 120 KPa^−1^, <0.5 KPa) and reduce the detection limit (0.88 → 0.41 Pa). This is due to the photoelectric effect and the good photothermal conversion efficiency of PPy for certain wavelengths of light. This light control strategy combined with the biological structure is a good use of the special scenario conditions of outdoor sports and has been well used in sports monitoring (Figure 13a).

Good posture is necessary both for aesthetics and for conforming to the body structure to protect the body. Sensors designed to monitor strain and vibration are functionally natural tools for posture monitoring [281]. Inspired by the epidermal tissue structure of human skin, Pang fabricated a pressure sensor with a biomimetic spinosum (RDS) microstructure [282]. Using frosted paper as a template and graphene as a sensing material, pressure sensing over a large linear range can be achieved. Due to the interlacing effect between the RDS layer and the uniform graphene coating, the pressure sensor can be used for gait monitoring under motion. The sensor under gait monitoring needs to be subjected to the pressure of the full body weight, which makes demands on the tolerance of the sensor, especially the surface wetting that occurs after prolonged contact separation cycles of the active layer. To overcome this challenge, Ye developed a self-powered flexible sensor based on solid–liquid frictional electrical nanogenerators [283]. The sensor encapsulates a highly flexible liquid metal in an embedded Ecoflex surface in a simulated sharkskin-like microstructure. This unique surface morphology renders the triboelectric layer hydrophobic, preventing adhesion of the liquid metal under pressure during sensing, which is favorable for device lifetime and reliability, as well as enabling highly sensitive real-time monitoring and long-term signal stabilization (Figure 13b). Inspired by squid skin color modulation via muscle contraction/relaxation, Lin proposed a class of dual-channel sensing systems capable of simultaneously implementing strain-acting electronic and visual optical signal responses [284]. The sensing laminate contains interbonded red fluorescent hydrogels and thin films of polydimethylsiloxane and carbon nanotubes (CNTs). The microscopic CNTs films form a network of microcracks and are densely stacked through a stretch–release process. The hydrogel laminate achieves simultaneous fluorescence color and resistance changes to provide motion state sensing along with visual feedback (Figure 13c).

#### 3.3.3. Sleep Monitoring

**Figure 14:**
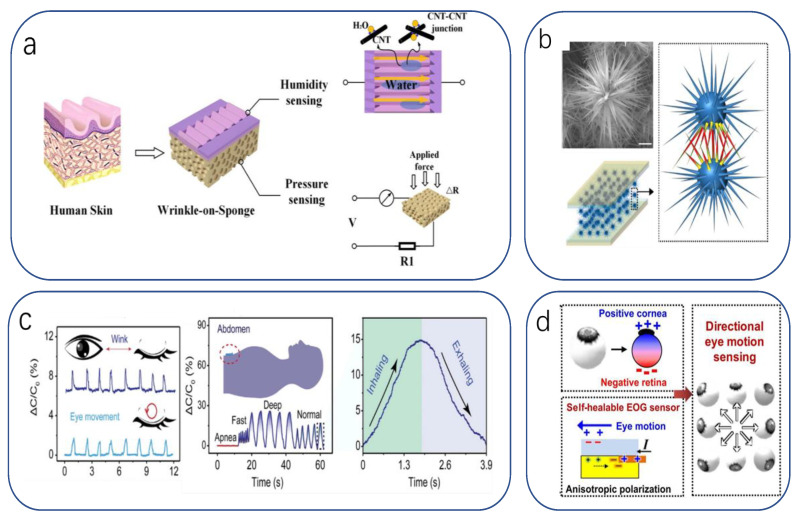
(**a**) Composite structure of a mature human skin-inspired CNT-PDMS: wrinkles at the top and porous sponge at the bottom [13]. Copyright 2019, American Chemical Society. (**b**) Top left: high magnification SEM image of a SUSM. Scale bar, 1 µm. Bottom left: schematic diagram of a sensor device made of a SUSM film sandwiched between two electrodes. Right: resistance modulation of the local spinal site induced by mechanical stimulation [288]. Copyright 2018, Springer Nature. (**c**) Capacitive response during subtle blinking and eye movements. The capacitance change signal of abdominal movements during monitoring and the complete respiratory waveform containing inspiratory and expiratory processes derived from abdominal movement detection [290]. Copyright 2022, John Wiley and sons. (**d**) Self-powered and self-healing frictional electrical sensor with tactile orientation sensitivity for electrooculogram monitoring [291]. Copyright 2022, Elsevier.

A good night’s sleep is the cornerstone of maintaining physical and mental health. However, hundreds of millions of people around the world suffer from a variety of sleep disorders that severely impact their personal work and health. Polysomnography (PSG) is the most commonly used sleep monitoring technique today, but it is complex, invasive, and expensive. Current noninvasive monitoring techniques [285,286] do not allow for high sensitivity, multiparameter monitoring, and comfort at the same time. For this reason, Miao’s team [13] fabricated a resistive sensor with a combination of two functional layers of flexible pressure–humidity, mimicking the inner–outer epidermal structure. The bottom layer is a pressure sensor with a porous structure, and the top layer is a moisture sensor with a wrinkled structure (Figure 14a). In the application scenario, the sensor integrated with the mask is worn to detect breathing: fast breathing has a higher frequency of resistance changes than slow breathing, and the curve of resistance changes is more regular. Similarly, the faster the breathing, the more frequently the humidity changes, especially in a confined environment such as a mask. From this, we can determine the state of sleep entry. Human respiration rate can also be monitored by electronic skin, where the main source of the signal is the moisture stimulation of breathing, and the sensing mechanism is based on the difference in proton and ion conductivity between low and high humidity conditions [287]. 

Blood pressure changes also need to be monitored during sleep. The signals of most physiological indicators of the human body are relatively weaker during sleep than during wakefulness, so a sensor with high static monitoring capability is needed instead. Sea urchin-shaped microparticles (SUSMs), similar to biological brushes, were used as the active material for the sensor by Yin et al. [288]. The active material, coated with PDMS on the outside, was placed in the center of the electrode material. It was demonstrated that the sensor was able to reach a lower limit of static pressure detection of 0.015 Pa, which is a rather low value compared to the other bionic pressure sensors in this paper, with a sensitivity of 121 kpa^−1^ and a response time of 7 ms, passing 2000 stress cycles (Figure 14b). Traditionally, sensors use hydrogels that are less sensitive and accurate in low microsignal acquisition for sleep. Inspired by the ordered crystalline regions of human muscle tissue and the microstructure of sensory receptors in epidermal tissue, Jia fabricated a bionic polyampholyte hydrogel with fatigue fracture resistance by a one-step copolymerization method, and like most pressure sensors under microstructure, the microstructure design of the hydrogel with random height distribution increases the contact area and sensing sensitivity in response to weak stimuli up to 1. 11 kPa^−1^, which is 18 times more sensitive than the flat hydrogel, although not as good as the data from the former work [289]. Jing Liu’s team developed a multifunctional cellulose-based hydrogel to fabricate a dual-mode resistive-capacitive integrated skin hydrogel sensor [290]. The ability to monitor respiratory rate, stasis, and eye oscillation during sleep (the eye has different movement patterns at different sleep stages) fulfills the application scenario of monitoring obstructive sleep apnea conditions in daily life (Figure 14c). Inspired by the principle of eye movement, KIM proposes a self-healing polymer-based frictional electrical device with anisotropic force sensitivity, in which the silver nanowire (AgNW) composite gel layer is a multilayer dielectric structure. The arrangement of AgNW in the composite gel enhances the sensitivity and anisotropy of the sensor in the direction of the force, thus allowing the discrimination of eight eye movement directions. Additionally, because it allows the detection of low-pressure motion at pressures below 100 Pa, the sensor can also monitor extremely weak blood pulse waves in the temporal artery around the human eye in applications where the sensor is used for electrooculography [291] (Figure 14d).

#### 3.3.4. Health Monitoring of Special Populations

**Figure 15:**
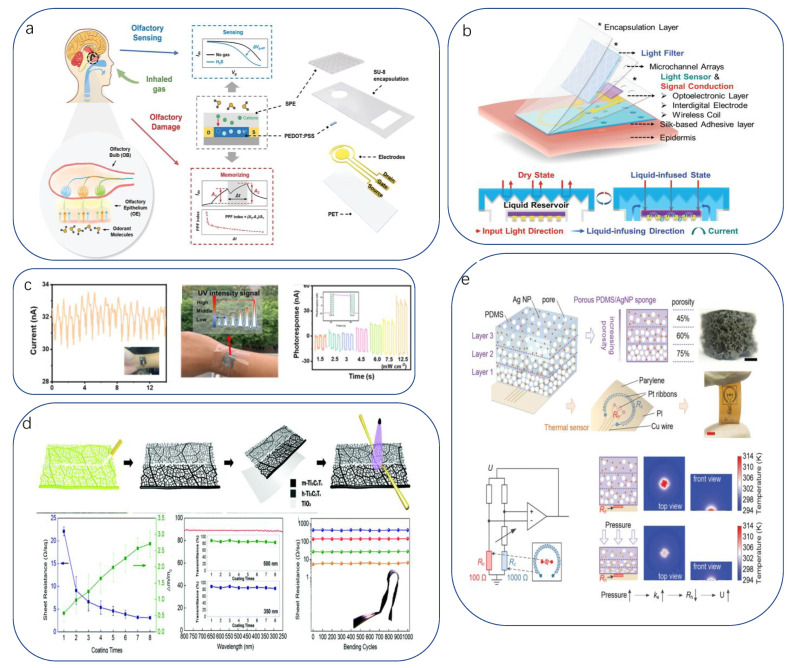
(**a**) Schematic diagram of flexible and bionic olfactory synapses based on organic electrochemical transistors (OECTs), synaptic devices mimicking olfactory sensing and potential damage toward gasotransmitter H_2_S. Schematic illustration of the working principle of the synaptic device (grey dashed line box). The shift of transfer curves of OECTs upon the exposure to H_2_S molecules enables gas sensing (blue dashed line box). The memorizing behavior of the synaptic device allows the imitation of olfactory damage due to the overexposure of H_2_S (red dashed line box). The bionic olfactory synapse device consists of a porous solid polymer electrolyte (SPE), a SU-8 encapsulation layer, a patterned PEDOT:PSS channel, drain, source and gate electrodes, and a flexible PET substrate [294]. Copyright 2023, John Wiley and sons. (**b**) Integrated wearable ASV mimicking a biological structure and consisting of several similar layers, including a customizable light filter, a flexible light sensor, and a wireless readout unit, the lower schematic illustration presents the working principle for microprism mode ASV [295]. Copyright 2020, John Wiley and sons. (**c**) Photograph of UV detection device and photocurrent for different doses of UV response [296]. Copyright 2022, American Chemical Society. (**d**) Schematic of the integration process of a leaf vein network-inspired UV photodetector, below is the relationship between the flake resistance and coating time, and the transmittance spectrum of the m-Ti3C2Tx blade electrode; Finally, the sheet resistance value of the blade electrode after bending 180° is obtained [297]. Copyright 2020, Royal Society of Chemistry. (**e**) Schematic of a pressure sensor consisting of a porous PDMS/AgNP sponge and a thermal sensor. The frame on the right is a photo of the sponge and thermal sensor, The black scalebar, 3 mm. The red scalebar, 5 mm. Below is a schematic diagram of the CTD regulating circuit. The steady state temperature field without pressure in the sensor is simulated by finite element analysis and the steady state temperature field in the sensor when the porous sponge encounters external pressure [302]. Copyright 2019, John Wiley and sons.

People in the chemical, machine shop, and transportation industries work in an environment full of health hazards, especially airborne colloidal contaminants and radiation. In the past, flexible chemical resistors have been used for the detection of dimethyl methylphosphonate (DMMP) and diisopropyl methylphosphonate (DIMP) [292], which are organophosphonate neural agents. In terms of flexible electronic skin sensors, devices with CNT as the active sensor substrate have been demonstrated to detect NH_3_, NO_2_, and EtOH [293]. Deng developed a flexible bionic olfactory synapse [294] that successfully enables wireless real-time monitoring of high concentrations of hydrogen sulfide gas and mimics the memory function of the biological nervous system (Figure 15a). Zhang discovered skin vision in many invertebrates, such as earthworms, jellyfish, and octopuses, due to the presence of photosensitive rod cells in the skin of these organisms, which enable optical perception and trigger colorimetric responses. Inspired by this, an artificial skin vision (ASV) wireless sensing device [295] was developed that does not require battery power and incorporates flexible optical sensing and optoelectronic elements to complete the process from light sensing and signal processing to expression, essentially mimicking the hierarchical structure and biological functions of rod cells in biological skin described above. The ASV device microfluidic channels and specially designed microprismatic optical filters are used to collect sweat and monitor intrinsic optical properties, respectively. The device has also been used to monitor the environment on the skin, such as UV exposure and degradation of sunscreen gels (Figure 15b). Yu’s team also proposed a three-dimensional flexible piezoresistive sensor for human motion and health monitoring, as well as UV detection [296]. Cotton fibers characterized by a three-dimensional cross-linked interlocking structure were soaked in graphene oxide (GO) and heated to reduce them to graphene cotton sheets, a process that enhanced the material’s electrical conductivity, tensile strength, and sweat resistance. Observation of homogeneous zinc oxide nanorods (ZnO NRs) grown in situ on graphene cotton sheets reveals a similarity to pine needles in terms of microstructure. The fabricated flexible sensors can be used to monitor outdoor UV light, and the preparation process embodies the significant potential for low synthesis cost, controllable fabrication size, and good performance (Figure 15c). However, for the efficiency of such UV detection devices whose light transmission is not high enough, in order to enhance the capture of UV light by the active layer to improve the sensitivity and efficiency, the easiest way is to make the device as transparent as possible. Jiaxin proposed a translucent MXene electrospun TiO_2_ thin film flexible UV photodetector inspired by the leaf vein network electrode [297]. The working function of the MXene electrode is performed by a different etching process, with a high light transmission of about 90% and a low resistance value of about 3 Ωsq^−1^ (Figure 15d).

Foot pressure is important information for biomechanics, health care, rehabilitation, and diagnosis, especially for patients with Parkinson’s disease or diabetic foot ulcers [298,299]. Based on the complex porous structure of the sponge, reversible compressibility, and light weight [300,301], Zhao and Zhu reported a flexible pressure sensor based on thermosensitive technology [302] that consists of a platinum ribbon and gradient porosity of a multilayer PDMS/AgNP sponge. Due to the gradient porosity, the deformation process is layer-by-layer, which contributes to high sensitivity, low detection limit (4.1 Pa), and wide measurement range (200 kPa). It is then integrated with a temperature sensor for temperature compensation to reduce the effect of thermal noise. Thanks to the wide measuring range, the sensor is able to monitor foot pressure, especially for different pedaling intensities (Figure 15e).

#### 3.3.5. Nutrition Monitoring

**Figure 16:**
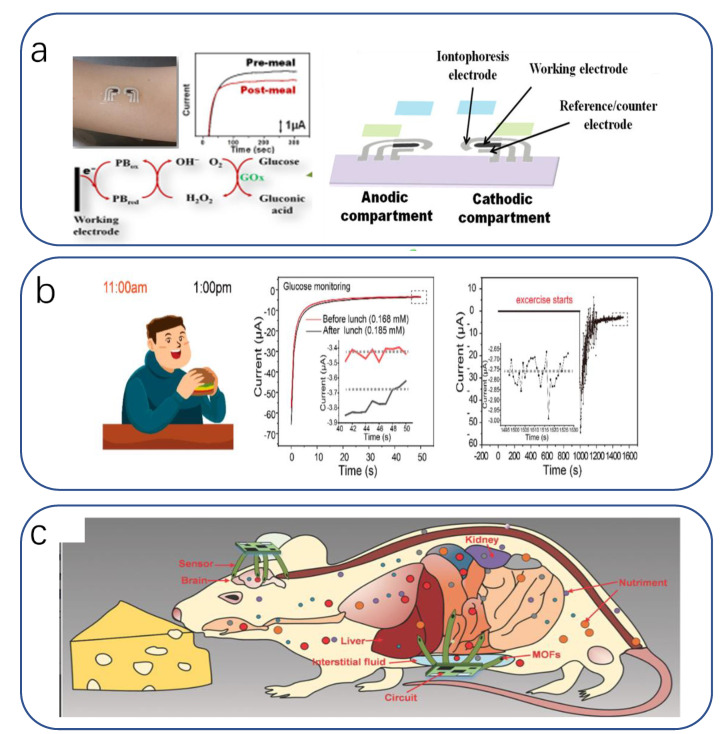
(**a**) Glucose sensor using interstitial fluid. Redox reactions of glucose lead to current changes [303]. Copyright 2015, American Chemical Society. (**b**) Real-time continuous sweat glucose monitoring using the glucose sensing patch InBody [306]. Copyright 2023, American Chemical Society. (**c**) Concept of a flexible MOF-modified sensor for nutrient sensing [307]. Copyright 2018, John Wiley and sons.

Whether in the pursuit of a healthier lifestyle or in the prevention and management of disease, people are aware that diet is a major management issue. For example, for diabetic patients, it is important to pay attention to the effect of diet on blood glucose, and Wang et al. fabricated a skin-like glucose sensor [303] (Figure 16a) to establish the accurate correlation between glucose concentration in ISF (interstitial fluid) and glucose concentration in blood. This skin sensor system for epidermal fluid analysis can be further developed in the future to monitor multiple biomarkers simultaneously, such as integrating the ISF sensor with the sweat sensor for multiple analyses (ethanol in sweat and glucose levels in ISF were measured simultaneously [304]). Observation of the bamboo leaf surface reveals alternating striated grooves (80–100 μm) and ridges (50–80 μm) on the surface [305]. For this purpose, Zhang designed a skin-like patch with bionic microfluidic channels fabricated using both stereolithography (SLA) 3D printing and femtosecond laser processing [306] for efficient sweat sensing. Striped grooves and ridges, similar to the surface structure of a bamboo leaf, are machined on the bottom of the microfluidic channel. The striped grooves enhance the capillary force in the channel, allowing sweat to flow rapidly during the wetting phase. The hydrophobic particle structure on the ridge top surface then enables fast flow in the channel even after the sweat infiltration session. The glucose concentration in the subject’s sweat is quickly measured in a human patch test (Figure 16b). Wei’s team proposed a tentacle-like multichannel sensor integrated with MOFs [307]. This sensor is capable of simultaneously measuring analyte distribution and transport in different organs and body sites. A high-throughput screen printer was first used to print multiple stacked layers of silver, carbon, and polymer insulator, and then the stacked layers were coated onto a polyethylene terephthalate (PET) substrate. The stacked layers have a sharp tip for easy implantation into organs and tissues. The sensor can be used to detect micronutrients such as ascorbic acid (AA), l-tryptophan (l-Trp), glycine, and glucose, which are closely involved in metabolic and physiological activities (Figure 16c).

## 4. Summary and Outlook

Bionics aims to study and simulate the structure, function, behavior, and regulatory mechanisms of living organisms, with implications for current engineering work in terms of providing new design concepts, operating principles, and system compositions, thus developing reliable, flexible, efficient, and economical technological systems in engineering. Bionic sensors are a novel field developed in recent years by the interpenetration of biomedicine and electronics, automation, and engineering. Biologically inspired sensors have improved sensitivity, selectivity, stability, and repeatability, while often being biocompatible and reproducible. Ideas are provided for realizing real-time health monitoring and personalized medical systems.

Most of the current research on flexible and wearable devices still focuses on the implementation of sensing functions for single or highly correlated bioinformatic indicators. However, real-life experience shows that most diseases require the joint diagnosis of multi-perspective physiological information and there is no doubt that multi-information monitoring of sensor devices is undoubtedly the answer we seek [12,66,154,308,309,310]. With the development of artificial intelligence and the need for complex medical monitoring, there will be an increasing focus on devices with higher integration and detection efficiency [47,75,311,312]. On the other hand, devices that are not washable and antibacterial are clearly detrimental to the application of medical and health-related activities. This challenge must be overcome by future bionic flexible sensors, even if they are designed with the development of individual monitoring by individual users in mind. The aesthetic properties of the devices also need to be improved and optimized. Nature can still provide us with suitable inspiration for this work, e.g., 3D-printed underwater superoleophobic shark skin, which is capable of self-cleaning in water through low-adhesion gliding force generation by magnetic nanofluid droplets [313].

Now, researchers are aware that with the often weak physiological signals, the slightest error can result in significant damage to signal integrity. In the monitoring process, water, vibration, and ill-fitting wear can affect signal acquisition, and thermal noise in the circuit, aging, and poor channel environment can also lead to incorrect processing of information. As the pace of intelligent medicine gradually approaches, the physiological signal from the human body to the monitoring terminal has more and more links, and how to ensure that monitoring remains reliable and accurate in continuous use or even extreme conditions is very necessary.

Taken together, current trends in academic research and market practice portend a leap forward in medical sensing. We believe that medical diagnosis, rehabilitation, and prevention will become efficient, fast, and accurate with innovations in artificial intelligence, big data, the Internet of Things, and wireless communication technologies. In order to produce more advanced and flexible devices that achieve consistent performance and operation stability, we also need to invest more resources in advanced manufacturing processes and higher-precision manufacturing equipment. All of this requires researchers in different fields, such as materials, mechanical, electronic, computer, biological, and medical, to continue their non-stop exploration and unremitting experimental efforts to truly make bionic flexible sensors an integral part of the body domain network for personalized medicine.

## Figures and Tables

**Table 1:** Summary of the materials and processes used in the demonstration of flexible materials preparation. (S: strain; P: pressure; T: temperature; H: humidity; R^2^: the linear degree of data linear fitting) [47].

Mechanism	Sensitivity (Linearity) (Detection Range)	Main Material	Structure	Fabrication	Refs
Piezoresistive/ thermoelectric	*S*; 220.8 (0–5%, R^2^ = 0.972), 1933.3 (53–62%, R^2^ = 0.991) *T*: 0.2 °C (resolution)	MXene-AgNWs/ PEDOT:PSS-TeNW	Brick/mortar film	Screen printing	[70]
Piezoresistive/ thermoelectric	*P*; 98.1% kPa^−1^ (1–130 kPa, *R*^2^ = 0.995), 98.1% kPa^−1^ (1–130 kPa, *R*^2^ = 0.995), *T*: 17.1 µV K^−1^	PANI/CNT/ PDMS	Foam	Synthesis/template	[178]
Piezo capacitive/ piezoresistive/ thermo resistive	*P*: 0.86 kPa^−1^ (0–100 kPa)	AgNW/ MWCNT/ PDMS/PET/ ITO	Ordered 3D pores	Microfluidic-assisted emulsion	[55]
Piezoelectric/ pyroelectric	*P*: 0.044 V kPa^−1^ *T*: 0.048 V °C^−1^	BaTiO_3_/Ag/ PDMS	Film/matrix	Magnetron sputtering/molding	[179]
Piezoelectric /pyroelectric /triboelectric	*P*: 0.092 V kPa^−1^ (10–50 kPa) *T*: 0.11 V °C^−1^ (10–45 °C, *R*^2^ = 0.982)	AgNW@PTFE/ graphene/ PVDF	Sandwich/ multipixel	Spin coating/laser/ screen-printing	[180]
Resistive	*S*: 33 (<50%) *P*: (0.66–1.2 MPa) *T*: (30–50 °C)	P3HT nanofibrils/ AuNP/ AgNW/ PDMS	Multilayer	Galvanic replacement process/drop casting	[181]
Resistive	*T*: (25–45 °C)	Cr/SiO_2_/ PDMS/PI/ SR	Multilayer	Thermal deposition/lift-off/spin-coating	[182]
Resistive	*P*: 1185.8 kPa^−1^ (0–5 kPa) *T*: (20–70 °C)	AgNP/ PEDOT:PSS/ SBS/PDMS	Multilayer/ Multi-pixel	Electrospinning/ drop-coating	[165]
Resistive	*S*: 0.45–2.08 (0–120%, *R*^2^ = 0.99) *T*: 0.55% (0–100 °C, *R*^2^ = 0.998)	MWCNT /PDMS	Fiber	One-step extrusion	[183]
Field effect transistor /thermo resistive	*T*: ≈0.89%/°C (25–45 °C) acceleration: 0.064, 0.057, 0.00% s^2^ m (5–12 m s^−2^)	Ag/PET/CNT/AgNP/PEDOT:PSS/Al_2_O_3_/ EGaIn/SR/ PDMS	kirigami	Printing/coating/ sputtering/ALD/EBE	[184]
Resistive/ thermoelectric/ capacitive	*P*: 0.41% kPa^−1^ (0–200 kPa) *T*: (30–55 °C) *H*: 0.08 (10–60%)	SNR/PI/ PDMS	Multilayer/ serpentine metal lines	Spin-on dopant/RIE/ Photo lithography/ etching	[153]
Resistive	*T*: 1.1%/°C (25–51 °C) *P*: 0.006%/Torr (793–868 Torr) Light: (6.5–310 mW cm^−2^) *H*: (10.9–64%)	PDA-modified graphene ink	Origami	Direct writing	[185]
Thermoelectric	*T*: (2–20 °C) Flow: 0–3 m s^−1^ Matter: 6 types *H*: 15–70% Power density: 3 µW cm^−2^	Bi_2_Te_2.8_Se_0.2_/ Bi_0.5_Te_3_Sb_1.5_/PI/parylene	Film	FPC/etching/CVD	[186]
Optical	Strain: 2–5 dB e^−1^ Bending: 7–24 dB cm^−1^ Pressing: 0.9–1.2 dB N^−1^ (2–5 N)	PU/silicone elastomer	Core cladding fiber	Replica molding and laminating	[81]
Resistive (ionic conduction)	*T*: 2.14%/°C (20–35 °C), 0.944%/°C (35–52 °C), 0.471%/°C (52–70 °C) *H*: (45–85%)	Ionic PVA hydrogel/ glycerol	Film with kirigami	Freeze thawing	[187]
Resistive/ transistor	*S*: 21 (<50%) *P:* 0.04 kPa^−1^ (50 Pa) UV–vis: 5.2 × 10^10^ Jones *T*: 2.2% °C^−1^ NO_2_: 33.6% ppm^−1^	IGZO/CuO/ PEDOT:PSS/ IL/SEBS	Film	Synthesis/blow-spinning/ casting	[188]
